# From prediction to function: Current practices and challenges towards the functional characterization of type III effectors

**DOI:** 10.3389/fmicb.2023.1113442

**Published:** 2023-02-08

**Authors:** Joren De Ryck, Petra Van Damme, Sofie Goormachtig

**Affiliations:** ^1^Department of Plant Biotechnology and Bioinformatics, Ghent University, Ghent, Belgium; ^2^Center for Plant Systems Biology, VIB, Ghent, Belgium; ^3^iRIP Unit, Laboratory of Microbiology, Department of Biochemistry and Microbiology, Ghent University, Ghent, Belgium

**Keywords:** type III effector, *Ralstonia solanacearum*, prediction, functional characterization, immunity, pathogenicity

## Abstract

The type III secretion system (T3SS) is a well-studied pathogenicity determinant of many bacteria through which effectors (T3Es) are translocated into the host cell, where they exercise a wide range of functions to deceive the host cell’s immunity and to establish a niche. Here we look at the different approaches that are used to functionally characterize a T3E. Such approaches include host localization studies, virulence screenings, biochemical activity assays, and large-scale omics, such as transcriptomics, interactomics, and metabolomics, among others. By means of the phytopathogenic *Ralstonia solanacearum* species complex (RSSC) as a case study, the current advances of these methods will be explored, alongside the progress made in understanding effector biology. Data obtained by such complementary methods provide crucial information to comprehend the entire function of the effectome and will eventually lead to a better understanding of the phytopathogen, opening opportunities to tackle it.

## 1. Introduction

### 1.1. The perpetrator: The *Ralstonia* disease

*Ralstonia solanacearum* is a bacterial phytopathogen that poses serious threats to agriculture due to its broad host range and its worldwide distribution. The disease, commonly known as bacterial wilt or brown rot, has been reported in more than 65 countries, affecting more than 200 plant species from over 50 botanical families, among which various solanaceous crops, such as tomato (*Solanum lycopersicum*) and potato (*Solanum tuberosum*) and a wide range of ornamentals (Mansfield et al., 2012; European and Mediterranean Plant Protection Organization, 2023). For these reasons, it was ranked as the second most important bacterial phytopathogen in molecular plant pathology worldwide (Mansfield et al., 2012).

Classification of the genetically diverse *R. solanacearum* species complex (RSSC) has been very dynamic over the years. In recent literature, the main classification used has been the phylotype distinction, because it provides evolutionary relationships and is based on the similarities in the DNA sequences of the 16S-23S internally transcribed spacer region, besides the *hypersensitive response and pathogenesis B* (*hrpB*), *endoglucanase* (*egl*), and *mutator S (mutS)* genes (Guidot et al., 2007; Remenant et al., 2010). The RSSC has been classified into four different phylotypes, reflecting its geographic origin, namely phylotype I, II, III, and IV strains, originating from Asia, America, Africa, and Indonesia, Australia, and Japan, respectively. Phylotype II strains are further subclassified into IIa and IIb. A detailed comparison of the genomes by average nucleotide identity (ANI) from different phylotypes revealed that genetic distances between strains were large enough to consider a reclassification into three distinct species, namely one containing phylotype I and III strains, termed *R. pseudosolanacearum*, another species consisting of phylotype II strains, termed *R. solanacearum*, and a third species encompassing strains from phylotype IV, termed *R. syzygii* (Remenant et al., 2010).

Instead of the categorization into phylotypes, the RSSC classification into five different races reflects its host range and pathogenicity (Buddenhagen et al., 1962; Denny, 2006). Race 1 strains can infect tobacco (*Nicotiana tabacum*), tomato, potato, eggplant (*Solanum melongena*), and diploid banana (*Musa* sp.), preferring temperatures of 35–37°C, just like race 2 strains that can infect triploid banana plants and *Heliconia* spp. and are responsible for the so-called “Moko disease” of banana. As such, race 1 and 2 strains are more widespread in tropical, subtropical, and warm temperate areas, whereas race 3 strains with a more limited host range can infect potatoes, tomatoes and weeds at moderate temperatures (27°C). Race 4 and race 5 strains can infect ginger (*Zingiber officinale*) and mulberry trees (*Morus* sp.) in China, respectively.

*Ralstonia solanacearum* owes its success to its ability to survive for an extended period in water, soil, and plant debris. To locate potential host roots, the pathogen uses chemotaxis, by which external signals, such as root exudates, influence bacterial motility and direction. Upon recognition, the bacteria stick to the root surface with polysaccharides, adhesin proteins, and type IV pili. From lateral root emergence sites or natural wounds, bacteria enter the roots and move inward toward the xylem vessels where they start growing, and are then transported systemically, either along with the sap flow or by twitching motility, eventually obstructing the flow of xylem sap (Planas-Marquès et al., 2020). The result is wilting and ultimately death of the host, subsequently releasing the pathogen into the environment, ready for the next infection.

Just like many other Gram-negative bacteria, the RSSC employs a type III secretion system (T3SS) that functions as a molecular syringe, pumping an array of virulence factors, designated T3SS effectors (T3Es) into the host cells to hijack the cellular signaling and manipulate the host’s immunity, with the pathogen’s unique infection strategy as a consequence. In this manner, the plant response is ineffective in preventing the disease, while the bacteria use its host as replicative niche and nutrient source.

### 1.2. The motive: The role of type III effectors in immunity and host susceptibility

Plants possess a multilayered immune system to deal with neighboring organisms that aim at using the plant as a host for nutrients and/or niche. As a first line of defense, conserved microbially associated molecular patterns (MAMPs), such as flagellin or lipopolysaccharides, are detected by specific pattern recognition receptors (PRRs) expressed on the surface of host membranes, resulting in an innate immunity response. These receptors are either plasma membrane-bound receptor-like kinases (RLKs) or receptor-like proteins (RLPs) that control a signaling cascade once the respective MAMPs are recognized. This innate immune response is referred to as MAMP-triggered immunity (MTI) or, in the case of pathogens, pathogen-associated molecular pattern (PAMP)-triggered immunity (PTI). Recognition of microbial patterns results in different defense responses, such as callose deposition, oxidative burst, cell wall strengthening, and expression of pathogenesis-related proteins.

At the molecular level, various modes of actions have been attributed to T3Es, among which MTI pathway inhibition (Büttner, 2016), and nutrient acquisition (Xian et al., 2020). Many T3Es target PRRs to shut down host defense pathways. Multiple T3Es from the same pathogen can even target the same PRR. The phytopathogen *Pseudomonas syringae* pv. *tomato* (*Pto*) DC3000, for example, encodes four effectors (AvrPto, AvrPtoB, HopF2, and HopQ1) that all (in)directly target the same leucine-rich repeat (LRR)-RLK FLAGELLIN-SENSITIVE 2 (FLS2), responsible for detection of flagellin. Together, these T3Es block downstream the FLS2 signaling by (i) hindering interaction with its coreceptor BRASSINOSTEROID RECEPTOR-ASSOCIATED KINASE 1 (BAK1; AvrPto, AvrPtoB, and HopF2; Shan et al., 2008; Zhou et al., 2014), (ii) preventing phosphorylation of its downstream signaling component BOTRYTIS-INDUCED KINASE 1 (BIK1; AvrPto; Xiang et al., 2010), (iii) targeting FLS2 for degradation (AvrPto; Göhre et al., 2008), or (iv) suppressing FLS2 accumulation in a cytokinin-dependent fashion (HopQ1; Hann et al., 2014). AvrPto also associates with ELONGATION FACTOR TU RECEPTOR (EFR), the PRR recognizing the bacterial elongation factor Tu (EF-Tu), to block MTI (Xiang et al., 2008). Interestingly, expression of the *Arabidopsis thaliana* EFR gene in tomato and tobacco (*Nicotiana benthamiana*) enhances resistance to various pathogens, such as *Pseudomonas*, *Agrobacterium*, *Xanthomonas,* and *Ralstonia* (Lacombe et al., 2010). Thus, signaling pathways downstream of the EF-Tu recognition are probably conserved between plant species and are interesting targets for disease resistance engineering. Notably, flagellin does not represent a major defense elicitor in *R. solanacearum* cells (Pfund et al., 2004), since the presence of polymorphisms in *R. solanacearum* flg22 avoids detection by FLS receptors from multiple plants such as Arabidopsis and members of the *Solanaceae* family (Wei et al., 2020). This finding, further exemplifies the evolutionary arms race occurring at the level of MTI. Additionally, T3Es may alter plant metabolism to obtain nutrient sources. The *R. solanacearum* T3E RipI, for example, interacts with and promotes activity of host glutamate decarboxylases that catalyze the biosynthesis of gamma-aminobutyric acid, a plant non-proteinogenic amino acid that can be used as nutritional source by the pathogen (Xian et al., 2020).

Plants can also distinguish T3Es either directly or indirectly by means of resistance proteins, enhancing the immune response, designated the effector-triggered immunity (ETI). Many resistance proteins contain a nucleotide-binding site/leucine-rich repeat (NB-LRR) structure. In ETI, pathogen spread is prevented by a hypersensitivity response (HR), i.e., a rapid localized cell death induced by reactive oxygen species (ROS) at the infection site. In *A. thaliana* Wassilewskija-2 (Ws-2), the RESISTANCE TO *RALSTONIA SOLANACEARUM* 1 (RRS-1) and RESISTANCE TO *PSEUDOMONAS SYRINGAE* 4 (RPS-4) proteins work as a dual resistance protein complex that recognizes the T3E *Ralstonia*-injected protein P2 (RipP2) of *R. solanacearum* (Narusaka et al., 2009). Moreover, the paired immune receptors RRS-1/RPS4 expressed in tomato triggers resistance to *R. solanacearum* and *Pto* DC3000 (Narusaka et al., 2013). Subsequently, coevolution has allowed many pathogens to develop mechanisms to overcome this immunity, for instance through the production of new ETI-suppressing effectors (Block et al., 2014).

As single T3E mutants rarely show an altered phenotype, T3Es have been hypothesized to form a robust interconnected network, in which the effectors can be seen as nodes and the interaction between the effectors or their cellular partners as edges, providing the bacterium with a certain flexibility (Sanchez-Garrido et al., 2021). However, this network robustness is limited, because individual effectors can become essential when part of the network is perturbed (Ruano-Gallego et al., 2021). This “context-dependent effector essentiality” illustrates the codependency of effectors. Effector-effector interactions may be involved in shaping this interconnected network. These interactions can be either antagonistic, where effector function or recognition by the host is reduced or prohibited, or synergistic, where the interaction promotes effector activity (Urbanus et al., 2016). This extra layer of regulation, also referred to as metaeffector activity, provides the pathogen with additional strategies to establish host susceptibility (Martel et al., 2022). Therefore, investigation of T3Es, their interactions, and functions *in planta* in a context-dependent manner is an important research area to understand the coevolution of effector-host interactions and to discover potential plant targets for resistance breeding. Although extensively studied, adaptation of breeding strategies to prevent phytopathogen attacks are still lacking for the RSSC, mainly because of its worldwide geographic distribution and acclimatization, broad host range, ability to survive in soil and water for extended time periods, and killer potential.

Whereas some T3E functions have already been discovered for different phytopathogens, many are still unexplored. Although the RSSC encodes the highest number of T3Es of all bacterial phytopathogens reported to date, the functions of only a few *Ralstonia* effectors have been described, in contrast to the extensively characterized T3Es of other well-studied bacterial phytopathogens, such as *P. syringae* and *Xanthomonas* spp. (reviewed in Schwartz et al., 2015). The reasons for the lack of knowledge on T3Es from the RSSC are most probably the size of the type III effectome (the repertoire of T3Es from a microbe) and its extraordinary diverse host range (Hayward, 1991).

### 1.3. Beyond a reasonable doubt: Challenges to overcome by the characterization of *Ralstonia* T3Es

As aforementioned, the description of the type III effectome of phytopathogens is complicated by the high number of T3Es typically encoded by phytopathogens. Effectors are usually grouped into families, referring to vertically inherited effectors indicative of an ancestral horizontal gene acquisition. To date, 112 curated T3E families have been reported for 155 strains of the RSSC with an average of 46–71 T3Es per strain.^1^ This number is higher than that of other well-known bacterial phytopathogens, such as *Xanthomonas* and *P. syringae* that encode, on average, 30 T3Es per strain (Schwartz et al., 2015; http://www.pseudomonas-syringae.org/). Basically, the functional characterization of the effectome has been lagging because of the large number of strains in the RSSC, each with its unique set of T3Es. To simplify matters, an interesting route could be to investigate the T3Es shared between strains, or the “core” effectome, that possibly represent the T3Es required for the successful pathogen infection strategy. For example, eight T3Es have been shown to be shared across 84 strains from four different phylotypes (Sabbagh et al., 2019). Nonetheless, “unique” effectors identified in phytopathogenic bacteria might provide more insight into specific host adaptation mechanisms. For instance, 11 and 9 T3Es are unique to phylotype I and III and to phylotype II strains, respectively (Sabbagh et al., 2019).

Alternatively, instead of focusing on the similarities or differences in T3E composition between strains, T3Es shared between strains and able to infect the same host could be investigated. However, because the RSSC has a broad host range that is difficult to define, such an approach is often complex. Nevertheless, the genomes of 25 RSSC strains grouped in different pathovars based on their host specificity were compared (Cho et al., 2019) and analysis of the pan-genome orthologous group and T3E repertoires revealed that, in contrast to their nonpathogenic variants, the pathogenic strains of tomato, eggplant, and pepper (*Capsicum annuum*) shared 8, 7, and 34 T3Es, respectively, but also that the T3Es RipS3 and RipH3 were found only in tomato-pathogenic strains and RipAC exclusively found in eggplant-pathogenic strains (Cho et al., 2019). From a dataset of 102 T3E sequences from the RSSC, the genomes of 140 distinct *R. solanacearum* strains were scanned for the presence of T3E sequences and 29 T3Es were common and 15 T3Es unique between *Ralstonia* strains isolated from tomato and eggplant, indicating that, although many T3Es are shared, different effectors might be required for different hosts (Sabbagh et al., 2019), a concept generally proven for other model species as well (Lindeberg et al., 2009; Schwartz et al., 2015). As the host range of the RSSC is very diverse, additional studies on plant host-specific T3E repertoires are required to further unravel T3E-host interdependencies. Recent research focusing on T3Es of mammalian pathogens even show that distinct effector networks are required depending on the occupied environmental host niche and context (Chen et al., 2021; Ruano-Gallego et al., 2021). Furthermore, because *R. solanacearum* occupies different parts of the plant during colonization and disease progression (Planas-Marquès et al., 2020), an interesting approach could be to investigate effector expression in a temporal fashion.

These examples indicate that the effectome size, the large strain diversity, and the broad host range result in a vast number of effector-strain-host combinations, especially complicating T3E research for the RSSC. Focus on unique or core T3Es or on T3Es associated with certain hosts can help the researcher in selecting the T3E to investigate.

In this review, we will examine the different manners to typify a T3E and, additionally, the effectome, based on previously identified T3Es and in view of reported methodologies with the RSSC as a case study. Moreover, we will highlight the current challenges toward the full and functional characterization of the RSSC effectome.

## 2. Different forensic disciplines for the functional identification of a T3E

### 2.1. Laying the foundation of the investigation: T3E prediction

Different approaches to predict T3Es can be divided into genome-dependent and genome-independent methods: the former include various computational prediction programs by which new T3Es are expected based on available information on T3E sequences from well-known plant and animal microbes, whereas the latter rely not solely on available genetic information, but also enable the identification of effectors based on functional analysis.

The computational prediction methods use *in silico* information on the intrinsic genetic elements from previously identified T3Es, not necessarily of the same species, and protein features, such as (positional) amino acid representation or the presence of translocation signal sequences, etc. For instance, T3Es frequently display a high G/C content and are often acquired by horizontal gene transfer. Additionally, T3E expression is typically under the control of a master regulator, denoted by specific regions in the promoter. Specifically for the RSSC strains, a *hrp_II_* box motif (TTCGn16TTCG) in the promoter region (Cunnac et al., 2004), necessary for HrpB-dependent activation, is commonly used as a criterion for RSSC T3E prediction, alongside homology to known T3Es and the presence of N-terminal specific export patterns (Peeters et al., 2013). Approximately 75% of the identified T3Es from *R. solanacearum* strain GMI1000 contain such a *hrp_II_* box motif in the corresponding promoter region (Cunnac et al., 2004). Likewise, 102 T3E-encoding genes and 16 hypothetical T3E genes in 12 evolutionarily distant strains from the RSSC have been detected (Sabbagh et al., 2019). By means of information on intrinsic genetic properties, different online tools were built allowing users to “blast” a query to the T3E database or scan a genome from the RSSC for the presence of putative T3Es (https://iant.toulouse.inra.fr/bacteria/annotation/site/prj/T3Ev3/# -Tools). As a consequence, T3E sequences in a genome could be screened when a new *Ralstonia* genome had been sequenced. The tool was, for example, used to identify T3Es from four different strains of *R. solanacearum* isolated either from blueberries (*Vaccinium*), hybrid tea roses, rocktrumpet (*Mandevilla*), or African daisies (*Osteospermum*; Bocsanczy et al., 2022). Many other tools for T3E prediction as well as T3E databases are also available, but are not restricted to the RSSC (for instance, The EuroXanth DocuWiki: https://internet.myds.me/dokuwiki/doku.php?id=bacteria:t3e:software). Some examples of the T3E prediction tools listed are, among others, Effectidor, EffectiveT3, and T3SPs (Arnold et al., 2009; Yang et al., 2013; Wagner et al., 2022).

**Figure 1:**
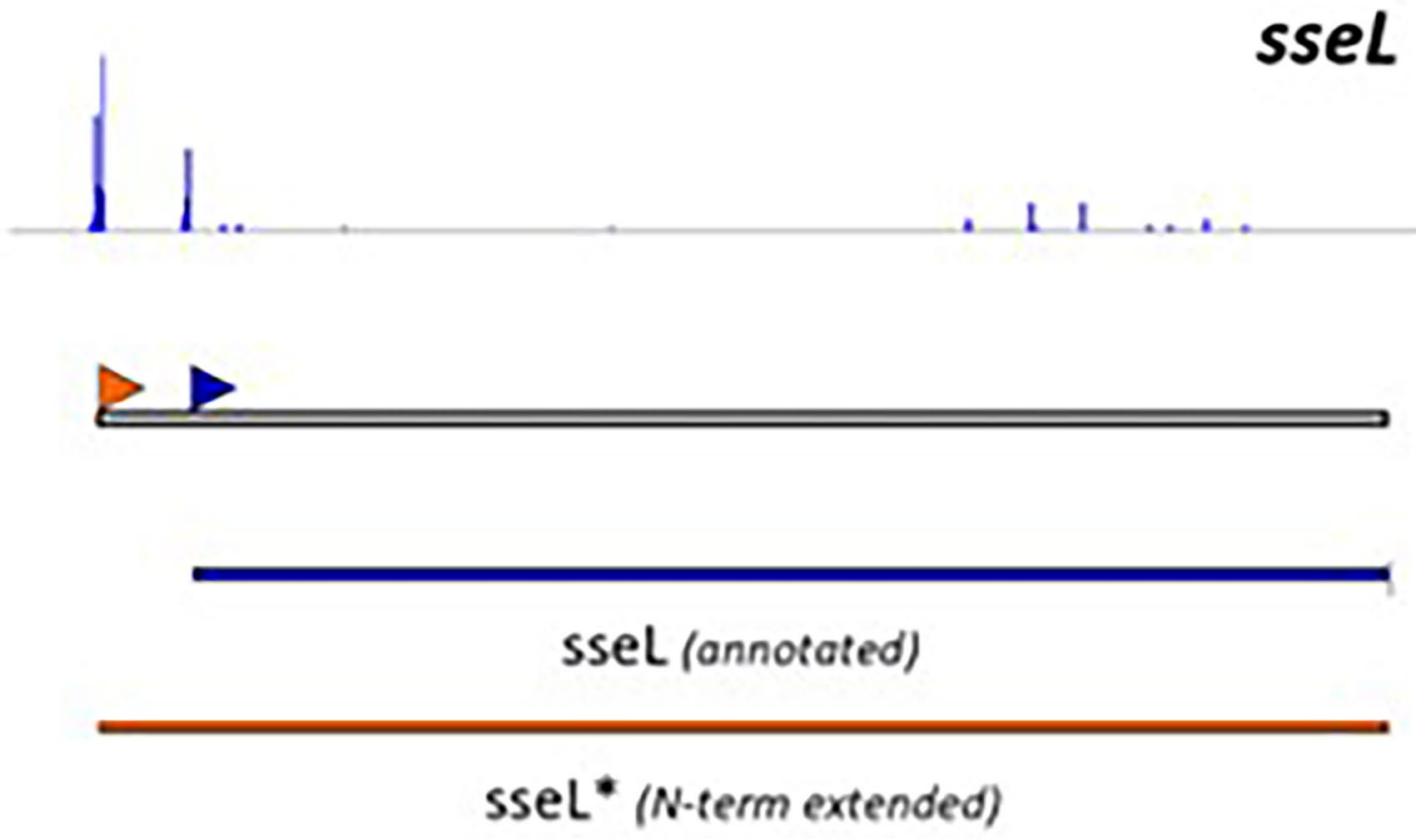
Retapamulin-assisted ribosome profiling (Ribo-RET) profile of *S. typhimurium* T3E SseL pointing to translation initiation at a newly identified upstream TIS. Bacterial ribosomes are specifically arrested at translation start sites by the antibiotic retapamulin. Here, translation initiation at a newly identified upstream TIS resulted in translation of an N-terminal extended SseL proteoform (Chromosome: 2392442–2393461). The annotated and newly discovered TISs are marked with a blue and orange arrowhead, respectively. Data from Fijalkowski et al. (2022).

Although useful, one should be aware that genome-dependent approaches can still produce false positive or false negative predictions. Indeed, not all characterized T3Es carry a conserved N-terminal secretion signal and many display a high sequence diversity, complicating homology-based database searches. Noteworthy, predictions obtained from different annotation tools concur moderately, even more so, when the accuracy of translation initiation site (TIS) predictions is considered. These findings are consistent with recent proteogenomic endeavors, revealing multiple translation initiation evidence that occurs at nonannotated start sites, besides showing extensive translation outside the annotated protein-coding regions. Start codon plurality within the same coding sequence, which - in the case of in-frame start codons - may result in translation of N-terminal proteoforms (i.e., molecular protein forms of a single gene originating from alternative TIS selection; Willems et al., 2022). As a representative example of T3E proteoform expression, an N-terminally extended proteoform of the *Salmonella enterica* subspecies *enterica* serovar Typhimurium (*S.* Typhimurium) deubiquitinase T3E SseL has been reported (Figure 1; Ndah et al., 2017; Fijalkowski et al., 2022), meaning that the correct annotation of the expressed open reading frame (ORF) is of critical importance, because it serves as the starting point for further follow-up studies.

Besides *in silico* prediction, tools based on functional characterization might be helpful in the search for new effectors. As the expression of many T3Es from the RSSC is regulated by the HrpB regulator, microarrays have been used to identify putative T3Es by comparing the expression of a wild type with a *hrpB*^−^ mutant strain. In this manner, 143 HrpB-upregulated genes were detected in *R. solanacearum* GMI1000 by means of a microarray consisting of 5,074 of the predicted coding sequences, thereby extending the repertoire with 26 new candidate T3Es and exemplifying that gene expression can also be used for the identification of T3E genes (Occhialini et al., 2005).

**Figure 2:**
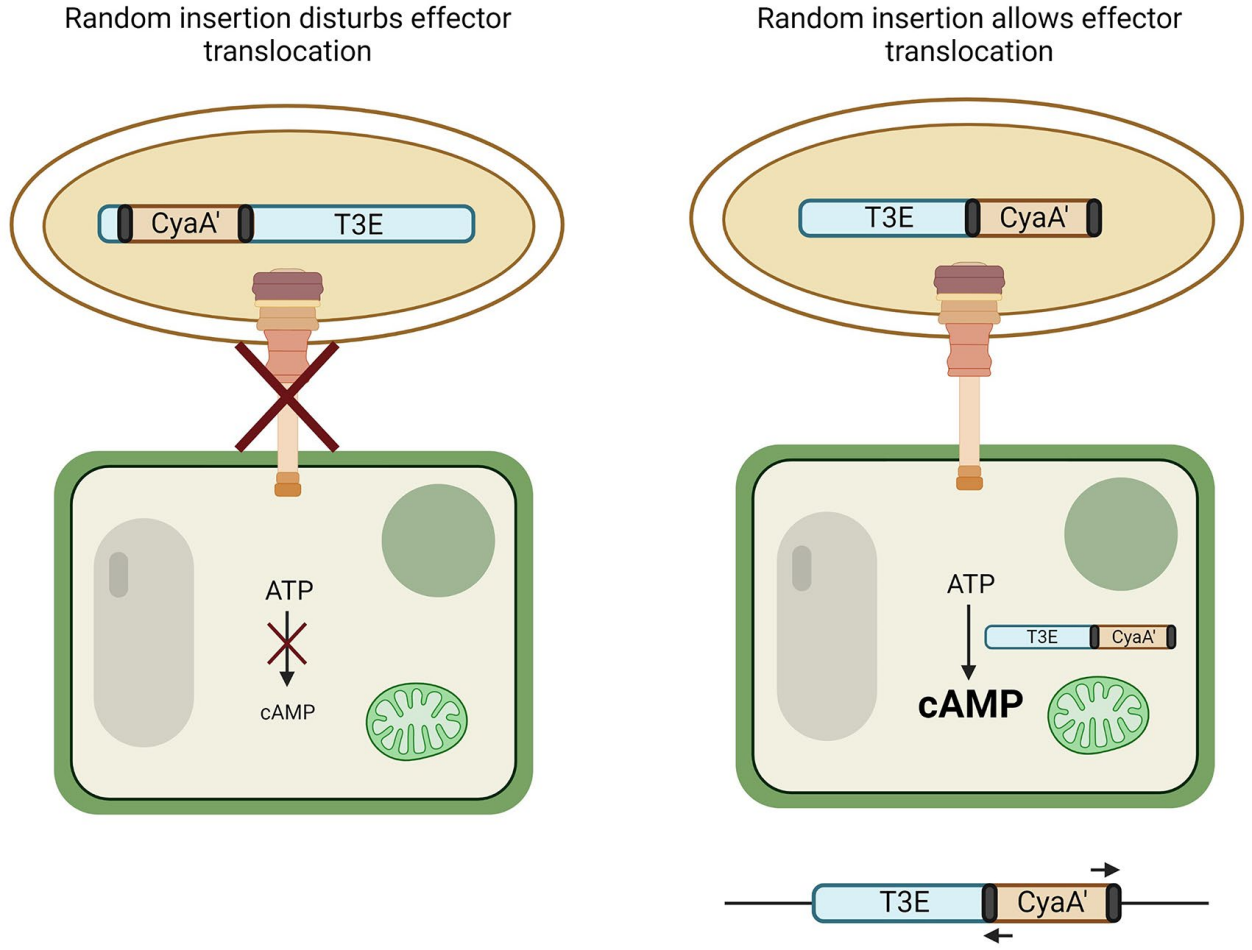
CyaA’ translocation assay to discover T3Es. By means of transformation, the N-terminal part of the calmodulin-dependent adenylate cyclase gene (*cyaA’*) is randomly inserted into the genome of the bacterium. If genomic insertion disturbs effector translocation to the plant cell *via* the T3SS, no change in cyclic AMP (cAMP) occurs (left). However, if random insertion allows translocation to the plant cell, CyaA’ converts ATP to cAMP (right). The genome of bacteria that induced plant cAMP levels can then be queried for the presence of an effector by use of primers binding to the transposon elements flanking the *cyaA’* gene (black).

Another genome-independent approach utilizes a truncated version of the adenylate cyclase domain from a calmodulin-dependent adenylate cyclase (CyaA’) of the cyclolysin toxin of *Bordetella pertussis*. This method requires, in contrast to genome-based T3E prediction, no prior knowledge of the genome or its encoded T3Es. Many T3Es from the RSSC have been discovered in this manner (Mukaihara and Tamura, 2009; Mukaihara et al., 2010) and a detailed protocol has been reported (Lonjon et al., 2018). In short, the CyaA’ reporter assay relies on the random bacterial genome insertion of an N-terminal-truncated *cyaA’* gene flanked by transposon sequences (Figure 2). If by chance the *cyaA*’ gene is inserted in-frame into an ORF encoding (part of) a T3E and if the insertion still permits translocation into the plant cell, the translocated effector-*cyaA*’ fusion leads to the accumulation of cyclic adenosyl monophosphate (cAMP) in the plant. Hence, because intrinsic cAMP levels in plant cells are low, an increase in cAMP would hint at the functional translocation of an effector-*cyaA*’-fusion protein. The upstream region of the transposon can then be queried from these mutants and checked for the presence of a *hrp_II_* box, supporting the discovery of an effector. Other methods to monitor effector translocation have been reviewed by Braet et al. (2022).

### 2.2. Unmasking the suspects: T3E validation by *in vitro* secretion or *in vivo* translocation

Once effectors have been predicted, functional validation is required. Secretion or translocation of putative T3Es should always be verified before further in-depth analysis. *In vitro* secretion of T3E candidates is often investigated by the analysis of a translational fusion of the T3E with an epitope tag. Under T3E secretion-inducing conditions in bacterial cultures, secreted T3Es can be detected in the bacterial supernatant by immunoblotting (Lohou et al., 2014). In view of the time needed for cloning and/or endogenous tagging, this analysis is time consuming when several effectors would be analyzed simultaneously. For a more global assessment of T3E secretomes, proteomics approaches can be used. In this manner, 35 T3Es were identified, among which the previously unidentified T3E RipBJ in the supernatant of *R. solanacearum* strain GMI1000, when compared to secretome profiles of T3SS-defective mutants (Lonjon et al., 2016).

To make T3E prediction and validation more comprehensive, a combined transcriptomic and proteomic approach can be selected, as done for the symbiotic bacterium *Bradyrhizobium vignae* strain ORS3257. Here, the transcriptome of a wild-type strain was compared to a T3E-transcriptional regulator mutant sampled under either T3E-inducing or noninducing conditions whereafter the supernatant culture of these strains was compared by shotgun proteomics. Combined with an *in silico* approach, 36 putative T3Es were identified in at least one of the approaches (Busset et al., 2021). This method could also work for the RSSC by comparison of the transcriptome and proteome of the wild type and a transcriptional regulator (*hrpB*) mutant strain.

Instead of the secretion assessment in culture media, *in vivo* translocation to the host plant cell of putative T3Es can be analyzed, although such an analysis might be complicated by the frequent nonsynchronous nature of infection and the time-dependent differences of T3E translocation, hence limiting the sensitivity of such *in vivo* methods. Nevertheless, CyaA’ reporters can, for example, also be used for the validation of T3E translocation into plant cells (Sory and Cornelis, 1994). So, for instance, the translocation into tobacco (*N. tabacum*) was validated for RipBJ by measuring cAMP levels of GMI1000 compared with a T3SS-impaired (*hrcV^−^*) mutant strain carrying RipBJ-CyaA’ (Lonjon et al., 2016).

### 2.3. Gathering leads: Online tools to aid in T3E characterization

Once a T3E has been identified, metadata can be gathered with several freely available online tools to help decide on further experimental steps. For example, it can be helpful to know beforehand at which subcellular host localization the translocated T3E is expected or whether the T3E has any relatedness with other better-defined T3Es. The presence of certain protein domains (e.g., enzymatic or protein interaction motifs/domains), potentially identified through structural similarity with other proteins, may assist in designing experiments for the characterization of the biochemical function and activity of the T3E under study. Here, we list some online tools that can be used for these purposes (Table 1). Although many other online prediction tools exist, they mostly provide comparable outputs.

**Table 1:** Freely available online tools to aid in characterizing a predicted T3E.

Tool	Description	Reference
***Subcellular localization***		
Localizer 1.0^2^	Scans the protein sequence for the presence of nuclear localization signals and transit peptides for chloroplast or mitochondria localization.	Sperschneider et al. (2017)	***3D protein structure***
	
Swiss-Model^1^^,^^2^	Builds a 3D protein structure assessment based on homology with other proteins and intrinsic amino acid properties.	Waterhouse et al. (2018)
I-TASSER^2^	Platform for automated protein structure and function prediction. Compares sequences with known structural templates and predicts function based by re-threading the 3D models through a protein function database.	Roy et al. (2010)
ModBase^1^	Database of Comparative Protein Structure Models	Pieper et al. (2011)
PredictProtein^2^	Provides structural (secondary structure, disordered regions, disulfide bridges) and functional (effect of point mutations, GO terms, subcellular localization and binding sites [protein, DNA, RNA]) annotations of the submitted amino acid sequence.	Bernhofer et al. (2021)
AlphaFold	A protein structure database without currently integration of sequence-based searches, but availability of the source code.	Senior et al. (2020)	DALI	Resource for protein structure comparison of the input protein structure with protein structures in the Protein Data Bank (PDB) or against a species subset from the AlphaFold database.
		Holm (2020)	***Protein domains***
	
InterPro^1^^,^^2^	Uses predictive models from different databases to provide domain predictions and functional analysis of proteins.	Mitchell et al. (2019)
CATH/Gene3D^1^^,^^2^	A tool that predicts 3D structure, protein evolution, protein function, conserved sites.	Dawson et al. (2017)
Motif scan^2^ (from MyHits)	Scans the sequence with a given window size to predict motifs compared to the genome background.	Pagni et al. (2007)
NCBI conserved domains^2^	Scans a protein or nucleotide sequence for conserved domains.	Marchler-Bauer et al. (2015)
Phyre2^2^	Performs homology searches (to build 3D models) and predicts ligand binding sites and the effect of amino acid variants.	Kelley et al. (2015)
BEAN 2.0	Besides a functional domain scan and disorder region annotation, also provides T3E prediction software and information on subcellular localization and builds a network highlighting relationships between T3Es.	Dong et al. (2015)
***Effect of amino acid modifications***			PROVEAN^2^
	Predicts whether single or multiple amino acid substitutions, deletions, and/or insertions abolish the function of a provided protein sequence.	Choi and Chan (2015)	***Phylogenomic databases***
			EggNOG^1^
	Provides orthologous groups, taxonomic profiles, and functional profiles.	Huerta-Cepas et al. (2016)	HOGENOM^1^
	Uses complete genomes from Ensembl and EnsemblGenomes (eukaryotes) and NCBI (bacteria and archaea) for building phylogenetic trees.	Penel et al. (2009)	OMA browser^1^^,^^2^
	Provides functional annotation, pairwise and groupwise orthologs, and synteny information.	Altenhoff et al. (2021)
***Protein–protein interactions***			EffectorK	Interactive tool that allows mining for published T3E-protein interactions in the *Arabidopsis* proteome.	González-Fuente et al. (2020)	STRING^1^	Functional protein association networks	Szklarczyk et al. (2019)
IntAct	Curated resource of molecular interactions, including T3E-host interactions.	del Toro et al. (2022)
***Posttranslational modifications***		
GPS^2^	Scans the protein sequence for potential posttranslational modification sites and provides disordered region prediction.	Xue et al. (2008)

1 Tool incorporated into UniProt.

2 Only the FASTA sequence required as input.

Some databases, such as InterPro or Uniprot, incorporate several different of the aforementioned prediction tools to provide an overview of the current knowledge on a certain entry, but they do not allow *do novo* sequence interpretations, albeit other tools (indicated by superscript 2 in Table 1) do look at intrinsic properties of the submitted sequence, whether or not supported by homology searches. Noteworthy, regardless of the program used, the output merely concerns predictions and requires experimental validation. For instance, application of the Phyre2 software to analyze the protein sequence of the *R. solanacearum* T3E RipAK provided information on putative functional domains and allowed the set-up of experiments by means of domain deletion mutants (Sun et al., 2017), resulting in the finding that the full-length RipAK is required for HR suppression in tobacco. Phyre2 was also used to investigate the T3E RipAY and revealed a high structural similarity with γ-glutamyl cyclotransferase (GGCT), as well as conserved catalytic key GGCT residues, leading to the discovery of the RipAY GGCT activity through mutational analyses of corresponding putative catalytic residues (Fujiwara et al., 2016).

An upcoming tool is AlphaFold and the more recent protein complex prediction algorithm AlphaFold-Multimer. AlphaFold uses a neural network to predict the 3D structure of individual protein chains based on information on the amino acid sequence, multiple sequence alignments, and homology to other proteins (Senior et al., 2020). Although currently it is still not possible to submit *de novo* protein sequences for 3D structure prediction in AlphaFold, this can be overcome by use of the source code available *via* Collabfold online. AlphaFold-Multimer was developed, extending AlphaFold to multiple chains as well (Evans et al., 2021), possibly allowing the mapping of effector-host protein interactions in the future. By means of DALI, a protein structure can be compared with protein structures in the Protein Data Bank (PDB) or with a species subset (such as *Arabidopsis*) in the AlphaFold database provided a protein structure has been predicted or defined and a PDB file of the protein is available (Holm, 2020). In this manner, it can become clear which other proteins share a similar 3D structure to use as a basis for further experiments.

### 2.4. Evidence collection: Experimental procedures to characterize a T3E

**Figure 3:**
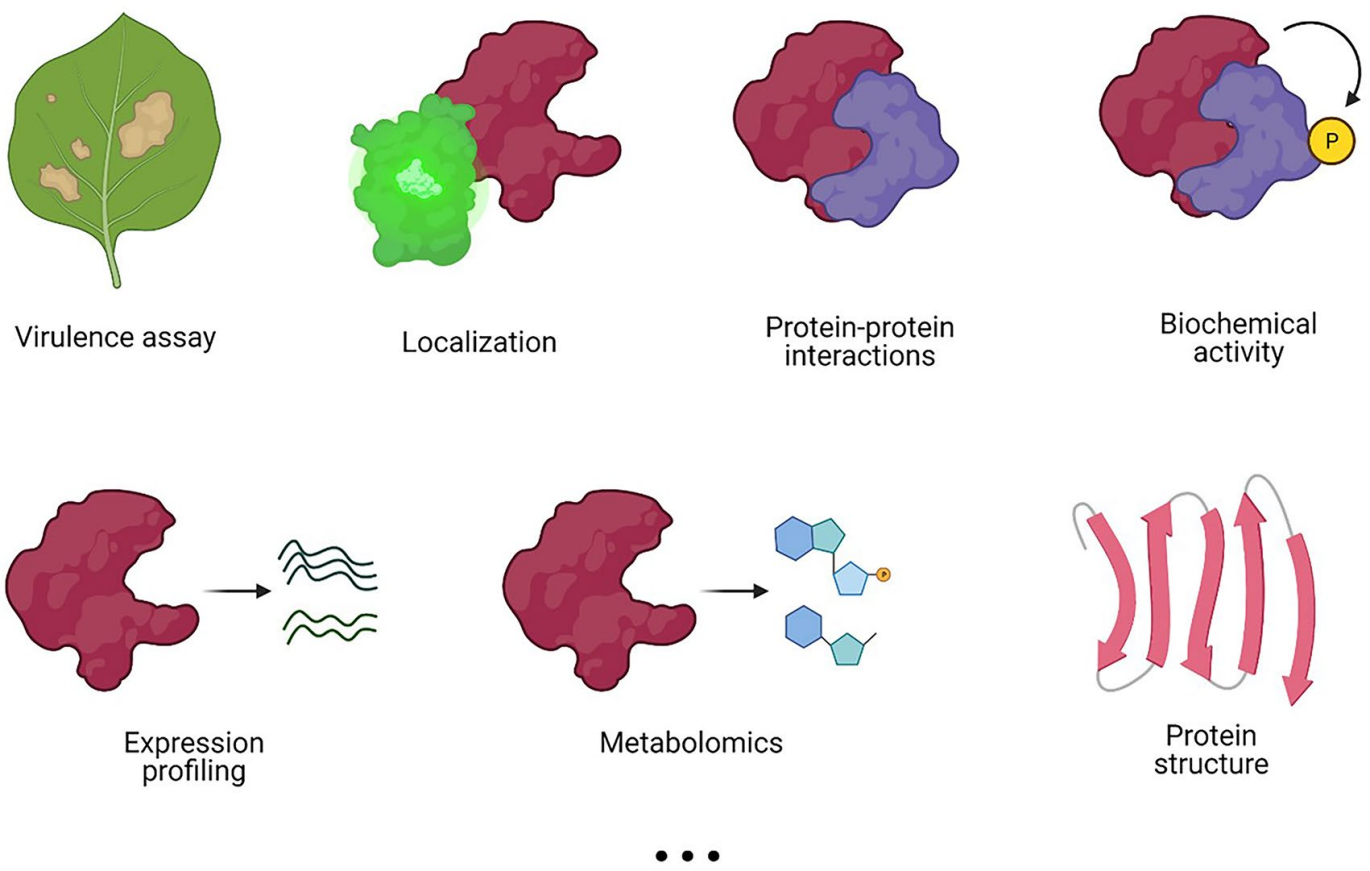
Different experimental methods to characterize a T3E. Various methods can be applied in a complementary fashion to elucidate T3E functioning. Commonly used experimental methods are listed, but this list is not exhaustive.

The method of choice for a functional characterization often depends on the field of interest, the background of the researcher, and the available resources. Nevertheless, different methods may offer complementary information to obtain a more complete view on T3E functioning (Figure 3). Whereas some methods, such as virulence assays, localization and/or protein structural analyses give no direct evidence of the effector’s function, they might provide a starting point for further characterization by other approaches, such as proteomics, biochemical activity assays, and metabolomics, among others (Figure 3).

To our knowledge, at present, only 14 T3Es from the RSSC have a known *in planta* function described and investigated with the aforementioned methods. In Table 2, the function(s) of these T3Es are summarized and as a representative example, in Table 3, the methods used to functionally characterize the T3E RipAB are listed. Other (extensively) characterized T3Es from the RSSC are listed together with the study methods in Supplementary Table 1. In this review, we will further discuss some of the commonly applied and upcoming state-of-the-art methods to functionally characterize T3Es.

**Table 2:** Known functions of T3Es from the *Ralstonia solanacearum* species complex.

T3E	Function	Reference
RipA5	Inhibitor of TOR signaling.	Popa et al. (2016)
RipAB	Interference with Ca^2+^-dependent gene expression.	Zheng et al. (2019)
	Interference with salicylic acid signaling by targeting TGA transcription factors.	Qi et al. (2022)
RipAC	Suppression of NLR-mediated SGT1-dependent immune responses.	Yu et al. (2020), Nakano et al. (2021)
	Targets plant E3 ubiquitin ligase PUB4.	Yu et al., (2022)
RipAK	Interaction with and inhibition of the activity of host catalases and pyruvate decarboxylases.	Sun et al. (2017), Wang et al. (2021)
RipAF1	ADP-ribosylation of host fibrillin FBN1.	Wu et al. (2022)
RipAY	Association with plant h-type thioredoxins and degradation of glutathione *in planta* to interfere with immune responses.	Sang et al. (2018)
		Fujiwara et al. (2016, 2020)
	Suppression of the RipE1-triggered immune response.	Sang et al. (2020)
RipB	Contributes to virulence and interferes with ROS production and cytokinin pathways.	Cao et al. (2022)
RipE1	Interference with jasmonate signaling.	Nakano and Mukaihara (2019), Sang et al. (2020)
	Interaction with and cleaving of the *Arabidopsis* Exo70B1.	Tsakiri et al. (2022)
RipI	Enhances the production of GABA to support nutrient acquisition during plant infection.	Xian et al., 2020
	Induces host defense by interaction with a bHLH93 transcription factor.	Zhuo et al. (2020)
RipN	Suppression of PTI and alteration of NADH/NAD^+^ levels in *Arabidopsis.*	Sun et al. (2019)
RipP2	Interaction with the R protein RRS1-R.	Deslandes et al. (2003)
	Autoacetylation of a lysine residue required for RRS1-R-mediated immunity.	Tasset et al. (2010)
	Acetylation of WRKY-TF to disrupt TF-DNA interaction	Le Roux et al. (2015)
	Binding with the putative resistance gene *RE-bw* in eggplant.	Xiou et al. (2015)
	Structural evidence for the RipP2-RRS1-R_WRKY_ interaction.	Zhang et al. (2017)
	Crystal structure of apo RipP2.	Xia et al. (2021)
	Interaction with PAD4 in an acetyl-transferase activity-dependent manner.	Huh (2022)
RipTAL	Activation of *ADC* genes to boost host polyamine levels.	Wu et al. (2019)
RipTPS	Management of production of plant trehalose-6-phosphate.	Poueymiro et al. (2014)
RipX	Suppression of the mitochondrial *atpA* gene.	Sun et al. (2020)

The methods used to characterize these T3Es can be found in Supplementary Table 1.

**Table 3:** Methods used to characterize RipAB and its putative function.

T3E	Function	Method	Goal	Model	Reference
RipAB	Interference with Ca^2+^-dependent gene expression	Virulence screening^1^	Revelation of the virulence contribution of core effectors.	Potato, tobacco, yeast	Zheng et al. (2019)
		Confocal microscopy^2^	*In planta* localization of RipAB.	Tobacco	
		Transcriptome analysis (RNA-seq)^1^	Effect of RipAB on plant processes.	Potato	
		qRT-PCR^2^	Validation of RNA-seq data.	Potato	
		Virulence assay^2^	ROS measurement.	Potato	
	Interference with salicylic acid signaling by targeting TGA transcription factors	Virulence assay^1^	Screen for T3Es containing nuclear localization signals that affect plant immune gene expression.	*Arabidopsis* protoplasts, *Arabidopsis*	Qi et al. (2022)
		Virulence assay^2^	Test for the effect of RipAB on ROS induction and MAPK activation.	Tobacco, *Arabidopsis* protoplasts, *Arabidopsis*	
		Confocal microscopy^2^	*In planta* localization of RipAB.	*Arabidopsis* protoplasts and *Arabidopsis*	
		Virulence assay	Test for RipAB overexpression or deletion resulting in more severe disease symptoms.	*Arabidopsis*, tomato	
		IP-MS^1^	Screen for plant protein interactors of RipAB.	Tobacco	
		BiFC, split-LUC, co-IP^2^	Validation of the IP-MS data.	Tobacco	
		GST/MBP pull-down assay^2^	Determination of direct or indirect binding of RipB with TGA TFs	*In vitro*	
		Virulence assay	Test for RipAB influence on SA-induced resistance and on RipAB virulence dependence on TGAs.	*Arabidopsis*	
		Transcriptome analysis (RNA-seq)^1^	Investigation of the influence of RipAB expression on SA-mediated gene expression.	*Arabidopsis*	
		RT-qPCR^2^	Validation of the RNA-seq data.	*Arabidopsis*	
		ChIP-PCR^1^	Search for the transcriptional targets of TGAs in the presence or absence of RipAB.	*Arabidopsis* protoplasts	
		Co-IP^2^	Test for RipAB interference with NPR1-TGA2 or with TGA2-RNA polymerase II protein interaction.	*Arabidopsis* protoplasts	
		ChIP-qPCR^2^	Test for RipAB interference with the ability of TGAs to recruit RNA polymerase II to the *PR1* promoter.	*Arabidopsis*	

Methods are not extensive and those relevant for the purpose of this review are listed. The putative function of other functionally characterized T3Es from the RSSC (RipA5, RipAC, RipAK, RipAF1, RipAY, RipB, RipE1, RipI, RipN, RipP2, RipTAL, RipTPS, and RipX) and the methods used to describe them can be found in Supplementary Table 1.

1 Screening method.

2 Validation method.

#### 2.4.1. Securing the crime scene: Subcellular localization

A reporter-tagged T3E is often expressed *in planta* to check full-length expression of the fusion construct and, additionally, to provide information on its subcellular localization. For phytopathogens, the go-to transient expression system is tobacco infiltration with an *Agrobacterium tumefaciens* strain carrying a binary vector for the selection and expression of a tagged effector gene. Other *in planta* expression systems might also be used, such as expression in transgenic roots obtained through *A. rhizogenes* transformation or transgenic *A. tumefaciens*-transformed *A. thaliana* (Deslandes et al., 2003; Tasset et al., 2010; Ron et al., 2014; Sun et al., 2017). Lastly, infection with a bacterial mutant strain encoding an endogenously fluorescently tagged T3E (by means of allelic replacement or recombination) or transformed with an alike T3E expression construct can provide insight into host effector localization in a physiological setting. In the case of fluorescent or luminescent reporter expression constructs, confocal microscopy or bioluminescence microscopy, potentially supported by the expression of subcellular markers, has been utilized to localize T3Es in different subcellular compartments (Denne et al., 2021). Whereas no direct information on T3E functioning is obtained this way, it can provide contextual information for the interpretation of the results acquired from other methods applied. RipE1, for example, localizes to the cytoplasm and nucleus of *N. benthamiana* cells (Nakano and Mukaihara, 2019). A yeast 2-hybrid (Y2H) screen with a cDNA library revealed an interaction of RipE1 with JASMONATE-ZIM DOMAIN (JAZ) proteins, known to be predominantly localized in the cell nucleus, thus matching the subcellular RipE1 localization (Nakano and Mukaihara, 2019).

Whereas native delivery of the T3E reporter by the phytopathogen is desirable over its (over)expression in the host, native translocation is limited, for instance, by the size or mechanical stability (i.e., the ease of protein unfolding under force) of the reporter tag used. This restriction was shown in the context of the green fluorescent protein (GFP) that displayed a comparable thermodynamic stability, but a higher mechanical stability compared to two *Salmonella* effectors (SptP and SopE2), implying that mechanical instability enhances secretion through the T3SS (LeBlanc et al., 2021; Braet et al., 2022). Some efforts have been made to overcome the passage drawbacks through the T3SS. For instance, the T3E RipP2 from *R. solanacearum* had been cloned to the C-terminal β strand of GFP (GFP_11_), instead of the commonly used full-length 11 β barrel strand-consisting GFP (Henry et al., 2017). The complementary part of GFP (GFP_1-10_) was expressed under a constitutive promoter in transformed *Arabidopsis* plants. After native delivery of the RipP2-GFP_11_ protein upon infection and complementation with GFP_1-10_ expressed in a *ripP2*-deletion mutant, a GFP signal was observed in the nucleus of *Arabidopsis* cells (Henry et al., 2017), a methodology commonly referred to as split-GFP. Other methods to study T3E subcellular localization, whether or not by native delivery, have recently been extensively reviewed (O'Boyle et al., 2018; Braet et al., 2022).

For the RSSC, the subcellular host localization of many T3Es has been reported. Recently, subcellular localization of a subset of 19 T3Es was assessed in tomato leaves and hairy roots, underlining the diverse destinations of different T3Es, including cell periphery, nucleus, tonoplast, and peroxisomes (Denne et al., 2021) and highlighting the importance of *in planta* localization studies, rather than relying solely on *in silico* localization predictions. Interestingly, subcellular localization prediction tools were accurate when the T3E was predicted to localize in the nucleus or plasma membrane, but less accurate for observed organellar or cytoskeleton localizations.

#### 2.4.2. Conducting a primary survey: Virulence and effect on plant immunity

Many T3Es have been shown to be involved in avoidance or suppression of the innate immune response. This function can be analyzed through several assays, such as an infection assay, in which, in the case of *R. solanacearum*, wilting symptoms are compared between a wild type and a (multiple) T3E deletion mutant. For example, for *R. solanacearum* strain OE_1-1_, a multiple effector mutant lacking 42 T3Es was generated and the disease progression of infected tobacco plants measured in a virulence assay, revealing that some T3E families, such as the RipA T3E family members, had a higher impact on virulence than others, e.g., RipG or RipH T3E families (Lei et al., 2020). Instead of examining the effector virulence through effector deletion (loss-of-function), the involvement of single effectors can also be tested in a gain-of-function experiment in an effectorless polymutant of *P. syringae* DC3000 strain (Wei et al., 2018), as was done to study the effect of five core *Ralstonia* T3Es (RipAE, RipAQ, RipC1, RipU and RipW) on plant immune responses (Cong et al., 2022). Here, RipU was shown to reduce the ROS burst, and to upregulate MAPK cascades in tobacco leaves. In addition to disease progression measurement, bacterial enumeration and, thus, replication inside the host are also used as a proxy to estimate the influence of one or more T3Es on virulence (Deslandes et al., 2003; Sun et al., 2017; Yu et al., 2020).

The involvement of a T3E in virulence can also be investigated by overexpression of the T3E within plant cells and by determination of the effect on mechanisms known to be implicated in immunity. Most often the development of ensuing immunity-related effects is evaluated, for instance, by performing cell death assays in tobacco leaves with an overexpression construct after agro-infiltration, HR induction assessment, ion leakage assays, electrolyte leakage monitoring from leaves as a measure for the cell death severity, or ROS assays (Table 3; Sang et al., 2018, 2020; Nakano and Mukaihara, 2019; Yu et al., 2020). Suppression of PTI by a T3E can also be examined in a PTI inhibition assay, based on the principle that previous PTI induction by a nonpathogenic bacterium can dampen the localized ETI necrotic response elicited by a pathogenic bacterial strain. However, when the nonpathogenic bacterium delivers a T3E that suppresses PTI, an ETI necrotic response occurs where the pathogenic strain was infiltrated (Badel et al., 2013; Le Roux et al., 2015). Additionally, expression of PTI/ETI marker genes, such as *FLG22*-*INDUCED RECEPTOR-LIKE KINASE 1* (*FLK1*) or *PATHOGENESIS-RELATED GENE 1* (*PR1*), is also often monitored (for instance, by reverse transcription-quantitative polymerase chain reaction (qRT-PCR)). PTI marker expression levels are regularly compared between leaves treated with the flagellin-22 (flg22) peptide (a bacterial PAMP, positive control) and leaves expressing the T3E or a mutant version (Le Roux et al., 2015).

#### 2.4.3. Checking for fingerprints: T3E expression profiling and the effect on the host transcriptome

The impact of T3Es on the host transcriptome is nowadays frequently investigated by means of RNA-sequencing (RNA-seq), although previously, microarrays had been used as well. In addition, the spatiotemporal expression of T3Es can also provide necessary information on the infection process itself and on the putative function of the T3E.

For instance, the spatiotemporal expression of T3Es examined by an RNA-seq approach in potato (de Pedro-Jové et al., 2021), retrieved RNA from potato plants infected with the cold-adapted *R. solanacearum* strain UY_031_ at three different infection stages, namely at an early, middle, and late stage, from the apoplast, xylem of asymptotic plants, and xylem of wilted plants, respectively. After normalization for bacterial counts, the T3E expression generally became more prominent at the middle and late stages, challenging the view that T3Es would mainly be important at early infection stages, although sensitivity at the earliest stages of infection might still be (too) limited. Nevertheless, some T3Es were found to have exceptional expression patterns, such as RipD and RipAD, both expressed at all infection stages (de Pedro-Jové et al., 2021).

The host can be monitored alongside the pathogen’s transcriptome with dual RNA-seq. This approach was used to observe both the T3E of *R. solanacearum* strain Rs-SY1 and the pepper gene expression during infection (Du et al., 2021). Compared to noninfected pepper plants, many pepper genes were differentially expressed 1, 3, 5, and 7 days after inoculation. Among others, at all stages, the genome ontology (GO) terms were enriched in the upregulated differentially expressed genes “defense response to bacterium,” “ethylene biosynthetic process” and “isoprenoid biosynthetic process” and in the downregulated differentially expressed genes “photosynthesis, light harvesting” and “response to abiotic stimulus.” Only genes involved in “sesquiterpenoid and triterpenoid biosynthesis” were gradually upregulated with disease progression. After mining the genome of *R. solanacearum* Rs-SY1, 84 T3E coding sequences were predicted, of which only seven had a higher expression in early infected pepper hypocotyls than that in the control, i.e., nutrient agar-grown *R. solanacearum*, hinting at the potential involvement of these T3Es at the onset of the disease (Du et al., 2021). Additionally, because only 7 out of 84 predicted T3Es were differentially expressed, the question was raised whether the other T3Es might have a function at later infection stages.

A transcriptomic approach can also investigate one or more T3Es under controlled expression instead of the broad T3E expression profiles during infection. The effect of T3E expression on transcriptomic profiles was studied in yeast cells ectopically expressing the T3E RipA5 (Popa et al., 2016). After measuring the genome-wide transcriptomic changes at several timepoints (2, 4, and 6 h) after induction of the RipA5 expression by means of DNA microarrays, 319 and 447 genes were discovered that were at least 2-fold induced and repressed, among which many nitrogen catabolite repression genes and ribosomal protein-encoding genes and genes involved in ribosome biogenesis, respectively. Intriguingly, this expression profile was fairly similar to that of rapamycin-treated yeast, a known inhibitor of the TOR COMPLEX 1 (TORC1) pathway. After some follow-up experiments, RipA5 could be shown to impact the TOR pathway *in planta* by acting as a TOR inhibitor in yeast and plant cells (Popa et al., 2016). A study on *in planta* function of the *R. solanacearum* T3E RipAB by RNA-seq (Zheng et al., 2019) revealed 417 differentially upregulated genes by comparison of three effector-expressing potato lines versus a control cultivar. A GO term enrichment analysis pointed to the involvement of RipAB in Ca^2+^ signaling (Zheng et al., 2019). In another experiment, RNA-seq carried out at different timepoints post inoculation (2 or 4 h) of *N. tabacum* cv. Xanthi leaves with wild-type *R. solanacearum* or with a *ripAK* deletion mutant identified regulated genes, including upregulation of photorespiration-related genes and downregulation of plant immunity-related genes, hinting at the involvement of RipAK in the modulation of early plant responses upon infection (Sun et al., 2017).

Transcription activator-like effectors (TALEs) also represent interesting targets for transcriptome-based analyses. TALEs represent a family of T3Es in the *Xanthomonas* genus, but also have one representative RSSC member, RipTAL1 (aka Brg11), that serve as transcription factors and activate the expression of certain virulence- or susceptibility-related genes by binding specific sites in target promoter regions. By differential RNA-seq of tomato plants infected for 24 h with *X. euvesicatoria* encoding either the *R. solanacearum* T3E RipTAL1 or RipTAL1 without its DNA-binding domain (DBD) under control of a constitutive promoter, the tomato *ARGININE DECARBOXYLASE* (*ADC*) genes *SlADC1* and *SlADC2* were identified as upregulated targets of wild-type RipTAL1 (Wu et al., 2019). Subsequently RipTAL1 was found to increase ADC activity and to boost plant polyamine levels, thereby potentially attenuating growth of *R. solanacearum* competitors.

#### 2.4.4. Interrogating witnesses: Proteomics and interactomics approaches to study effector functions

For the inspection of differing protein expression profiles, a shotgun proteomics approach can be taken. Such a method was used to monitor proteome changes in early and later stages of *P. syringae* infection (Fan et al., 2019). Similarly, it can be applied to investigate the effect of a microbe on the posttranslational modifications of the host (Walley et al., 2018).

However, these methods do not provide information on the direct function of a T3E. As many T3Es exert their function by binding with host proteins or metabolites, knowledge on their interactions is thus of fundamental importance. In this section, we will focus on different methods that can be used to elucidate effector-host protein–protein interactions (EH-PPI), each with their advantages and disadvantages as recently extensively reviewed (Struk et al., 2019; De Meyer et al., 2020).

Although certainly valuable, functional protein microarrays have rarely been used to examine PPIs, mainly due to the complexity and the cost of the microarray design. In a protein microarray, individual proteins are immobilized on a microarray and a purified protein of interest is applied, resulting in a readable signal. In this manner, a proteome array probed with 15,000 proteins (56% proteome coverage) has been designed for *Arabidopsis* (Popescu et al., 2007; Manohar et al., 2014). Even though microarrays allow the detection of weak PPIs, the native context is missing, resulting in high false positive and negative rates because of the absence of chaperones (and thus also correct folding), posttranslational modifications, etc. Nevertheless, the interaction between a large set of human proteins and several type IV effectors from *Legionella pneumophila* (Yu et al., 2015, 2018) was successfully studied using protein microarrays, but, to our knowledge, proteome microarrays to investigate the interaction between T3Es and plant proteins are yet to be reported.

More frequently, Y2H-sequencing (Y2H-seq) and mass spectrometry (MS)-based approaches, such as affinity purification (AP) or biotin identification (BioID) are used to identify EH-PPIs. In Y2H, a bait protein (such as a T3E) is fused to a DBD and the prey (such as plant protein) to an activation domain (AD) or vice versa. Upon direct interaction, the DBD and AD are brought together, resulting in the reconstitution of a transcription factor that drives the reporter gene expression, often a gene responsible for the production of an essential amino acid. Conventional Y2H is frequently used for PPI validation, but also as screen by means of cDNA libraries, as in the case of Y2H-seq. In Y2H-seq, the bait (for instance, a T3E-expressing) yeast strain is supertransformed with a cDNA prey library, allowing the discovery of new PPIs. The cDNA library can be obtained by cDNA synthesis from the RNA of, for example, *Arabidopsis* plants infected with the phytopathogen of interest to reflect the expression profile more closely upon infection. cDNA libraries should preferably represent the expression profile upon infection, but might also be made after treatment of the host plant with biotic stress-related phytohormones, such as jasmonate or salicylic acid. The ensuing yeast colonies after supertransformation and selection are then pooled, the extracted plasmid DNA sequenced, and the resulting sequencing reads mapped to the host genome (in this case *Arabidopsis*; Erffelinck et al., 2018). The results of a large-scale Y2H-seq screening experiment for T3Es from two distinct vascular phytopathogens (*R. solanacearum* and *X. campestris*) against a cDNA library of *Arabidopsis* (González-Fuente et al., 2020) have been made public *via* an online interactive database^2^ and show that T3Es from distinct pathogens share some interactors, commonly referred to as plant effector hubs, whereas others are specific to the pathogen. Although Y2H-seq is a high-throughput and relatively cheap method, it is also linked to high rates of false positives and negatives due to the yeast background, protein folding artefacts, underrepresentation of full-length prey proteins encoded in cDNA libraries -especially large proteins-, and membrane protein targets.

For these reasons, *in planta* methods, such as (tandem) AP/IP or BioID coupled to MS-based methods are often preferred. The increasing sensitivity of mass spectrometers allows label-free quantification (LFQ), thereby facilitating experimental setups. Postmetabolic labeling approaches, such as tandem mass tag (TMT) labeling, are commonly used for multiplexed quantification. A frequently used IP-MS method is GFP trapping, in which GFP is fused to the protein of interest and antibodies against GFP precipitate the interaction complex. In this manner, interaction partners of several T3Es have been identified, such as the h-type thioredoxin targets (NbTRX-h9, NbTRX-h10, NbTRX-h11, and NbTRX-h14) of the *R. solanacearum* T3E RipAY in tobacco plants (Sang et al., 2018). By contrast, BioID relies on the use of a promiscuous biotin ligase that biotinylates primary amines of vicinal proteins after biotin application. When fused to the bait of interest, the proxeome (proteins in the bait proximity) can be identified by streptavidin-based purification. Over the years, different versions of the biotin ligase have been engineered: *Escherichia coli* BirA* (R118G; 35.3 kDa), *Aquifex aeolicus* BioID2 (27 kDa), and *Bacillus subtilis* BASU (28 kDa; Choi-Rhee et al., 2004; Kim et al., 2016; Ramanathan et al., 2018). However, these biotin-dependent proximity labeling tags show slow kinetics, require long biotin labeling times, and thrive at an optimal temperature of 37°C, making them rather unsuited for usage in plants. Nevertheless, despite poor labeling kinetics, the BirA* tag was successfully applied in rice (*Oryza sativa*) protoplasts to study the rice transcription factor OsFD2 (Lin et al., 2017) and later in transgenic *Arabidopsis* to investigate the proxeome of HopF2, a T3E from *P. syringae* (Khan et al., 2018). An AP-MS to complement the BioID results revealed an overlap of 58% (11/19) of the putative targets identified, although some previously reported interactors of HopF2 were missed with the BioID approach, probably because of the inherent BioID limitations, such as unavailability of free lysine residues in interacting preys, etc. (Khan et al., 2018). To overcome some of the challenges of the BirA* tag, such as a long labeling time, high biotin concentrations, and optimal temperature of 37°C, two new versions were created that enabled more efficient *in planta* proximity labeling. By a directed evolution-based approach, TurboID and its shorter version miniTurboID were established, allowing faster labeling and working temperatures around 30°C or less (Branon et al., 2018). Compared to the other proximity-labeling tags, TurboID was the most effective in different plant models, working at temperatures ranging from 22 to 28°C, and necessitating the addition of biotin for shorter periods of time (~2 h) for the efficient capture of plasma membrane interactomes (Arora et al., 2020). TurboID was, for example, used in tomato hairy root cultures to investigate the *R. rhizogenes* effector RolB, leading to the identification of TOPLESS and Novel Interactor of JAZ (NINJA) as direct interactors of RolB (Gryffroy et al., 2023). UltraID (19.7 kDa), a directed evolution variant of BioID2, has recently been developed, requiring even shorter labeling times, and showing less background biotinylation in the absence of exogenous biotin (Kubitz et al., 2022) in the case of mammalian cell culture, yeast, and bacteria. The use of ultraID *in planta*, however, has not been reported yet.

When these EH-PPI methods are used, a list of putative interactors is acquired. The read count or the peptide count/protein intensity (LFQ) and the expression ratios obtained from the Y2H-seq or MS-based methods can be indicative for the reliability (and proximity) of a certain hit. Although Y2H-seq, AP-MS, and BioID are complementary, the overlap of identified proteins is rather limited, mainly, as mentioned, because of the method inherent limitations and the fact that Y2H returns binary interactions, whereas AP-MS and BioID also can provide proximal interactions. Therefore, further validation of direct and indirect interactions remains necessary. For direct interactions, the highest scoring hits can be verified with conventional Y2H, co-immunoprecipitation (Co-IP), Förster Resonance Energy Transfer-Fluorescence-lifetime imaging microscopy (FRET-FLIM), bimolecular fluorescence complementation (BiFC), or split-luciferase, each with their advantages and disadvantages (Struk et al., 2019). Validation of indirect interactions requires alternative follow-up studies. Recently, a new validation technique for application in plants, designated knocksideways (KSP) is based on the heterodimerization of two domains, the human FK506-binding protein (FKBP) and the FKBP12-rapamycin-binding domain of mTOR (FRB) upon the addition of rapamycin (Winkler et al., 2021). FKBP is typically fused to the bait protein and FRB to an organellar marker of choice that differs from the subcellular localization of the bait protein. Upon rapamycin treatment, both domains reconstitute at the chosen organelle, unless the delocalization of the bait protein is hindered by interaction with a prey protein of interest. All three studied proteins are labeled with different fluorophores, so the (change in) localization can be monitored. An image analysis script was developed to quantify the interactions. Thus, KSP allows *in planta* localization of bait and prey fluorophores as well as validation of multiprotein interactions and quantification of the interaction.

#### 2.4.5. Criminal profiling: Metabolomics

Besides their interaction with host proteins and/or alteration of host RNA levels, T3Es can also modify the host metabolism. Metabolomic approaches are frequently useful to describe microbe-host interactions. For example, an untargeted gas chromatography/MS metabolomics experiment revealed that 22 metabolites were enriched in the xylem upon infection of tomato plants with *R. solanacearum*, of which eight could be used as sole carbon or nitrogen source (Lowe-Power et al., 2018). Although in microbe-host metabolomic experiments, it is often challenging to determine which organism produces the identified metabolite, *R. solanacearum* was shown to be responsible for the production of putrescine, one of the 22 enriched metabolites found, because the pathogen harbors the corresponding biosynthesis and export genes. Putrescine was required for proliferation inside the xylem and an increased disease progression (Lowe-Power et al., 2018). Several attempts have been made to distinguish more easily the host from the microbe metabolites (Allwood et al., 2010; Pang et al., 2018). By labeling the metabolites from *P. syringae* DC3000 with stable heavy isotopes, *Arabidopsis* guard cell metabolites could be separated from microbial metabolites (Pang et al., 2018). This approach also allowed the separation of metabolic profiles at different timepoints after infection. Nevertheless, the role of T3Es in shaping the host metabolite landscape is still poorly understood. The use of single (or multiple) T3E deletion mutants could be insightful to determine their individual (or combined) roles in the generation of metabolomic changes during infection, but very few untargeted metabolomics have been done for single phytobacterial T3Es thus far. One example, however, is coumaroyl tyramine, a metabolic compound produced in the phenylpropanoid pathway that accumulates by the T3E WtsE from the maize (*Zea mays*) pathogen *Pantoea stewartii* spp. *stewartia* (Asselin et al., 2015). This compound was identified through LC–MS/MS by comparing the metabolite profiles of maize seedling leaves infected either with the wild-type or with the *wtsE*-defective strain. Instead of a metabolomic approach, many more targeted approaches have been utilized to determine the effect of a T3E on specific host metabolites, as, for instance, accumulation of salicylic acid, jasmonic acid and glutathione by the *R. solanacearum* T3E RipE1 was previously reported (Sang et al., 2020). After the identification of accumulated/depleted metabolites caused by T3E expression, a biochemical activity assay could be set up to pinpoint whether this T3E is directly (enzymatically) responsible for the accumulation/depletion of the compound.

#### 2.4.6. Determining the *modus operandi*: Biochemical activity assays

Driven by predictions and gathered data, experiments can be set up to pinpoint the exact function of the T3E of interest *in planta*. In the case of predicted protein domains with a known enzymatic function, targeted biochemical assays can be carried out. For instance, a ChaC domain that displays a GGCT activity in yeast and mammalian cells, was also found in the RipAY protein sequence and prompted the analysis of its GGCT activity and its ability to degrade glutathione (Fujiwara et al., 2016), revealing that RipAY had a robust GGCT activity in the presence of yeast or plant thioredoxins. Later on, RipAY was shown to interact with h-type thioredoxins *in planta* (Sang et al., 2018). Similarly, the discovery of the RipP2 acylation (i.e., (auto)acetylation) activity was steered by its homology to the YopJ-like family of effectors with a wide range of activities, such as (de-)sumoylation (AvrRxv and AvrXv4 from *X. campestris* pv. *vesicatoria*), de-ubiquitination and acetylation (YopJ from *Yersinia* spp.; Mukherjee et al., 2006, 2007; Roden et al., 2007), leading to the determination of the exact acetylation targets of RipP2 (Tasset et al., 2010; Le Roux et al., 2015). A third example is the *R. solanacearum* T3E RipTPS that presents homology with proteins harboring trehalose-6-phosphate (T6P) synthase (TPS) activity and really catalyzes T6P synthesis in yeast cells (Poueymiro et al., 2014). Besides relying on homology of protein domains to design follow-up experiments, known or newly identified EH-PPIs can also inform the selection of biochemical activity assays. For example, a catalase activity assay revealed that RipAK interacts with host catalases and pyruvate decarboxylases, thereby inhibiting host catalase activity and suppressing the plant’s HR (Sun et al., 2017; Wang et al., 2021).

#### 2.4.7. Generating a facial composite: T3E and T3E-host complexed protein structures

The examples mentioned above indicated that information on the protein structure can aid successful functional characterization of T3Es. Determination of the structure of a protein (complex) is often a challenging task (Fujiwara et al., 2020). Nevertheless, the protein structures of some T3Es have been elucidated. So, the structure of *Pto* DC3000 T3E AvrPto has been solved in solution by nucleic magnetic resonance (NMR) spectroscopy (Lee et al., 2004) and the crystal structure of AvrB was determined by single wavelength anomalous dispersion (SAD; Wulf et al., 2004). For the RSSC, only the crystal structures of RipP2 (apo or in complex) have been described (Xia et al., 2021). Complexed protein structures are also interesting for the characterization of the protein function. In one case, the crystal structure was determined for RipP2, a known acetyltransferase, in complex with its host cofactor inositol hexaphosphate (IP_6_), acetyl-coenzyme A (AcCoA), and the host resistance protein harboring a WRKY motif (RRS1-R_WRKY_; Zhang et al., 2017). The WRKYGQK motif of the RRS1-R_WRKY_ substrate drives RipP2 to a lysine residue in the acetylation active site pocket. Additionally, IP_6_ enhanced AcCoA and WRKY binding with RipP2, providing novel insights into the regulation of the RipP2 activity. To our knowledge, other RSSC T3E protein structures are still to be reported.

A potential solution for the challenging protein structure determination has recently been developed. The program, designated AlphaFold, relies on artificial intelligence to predict protein structures and has a high prediction accuracy (Senior et al., 2020). The AlphaFold database is currently vastly expanding, including additional proteomes and catalogued protein structures for even more accurate predictions. At the publication time, the AlphaFold source code is accessible online (Collabfold) and can be used to predict the protein structure of any protein of interest, as was done for the *Ralstonia* T3E RipE1 that harbors a cysteine protease domain (Tsakiri et al., 2022). More recently, the algorithm AlphaFold-Multimer has been created that could be used for the modelling of (effector-host protein) interactions (Evans et al., 2021).

## 3. Conclusion

**Figure 4:**
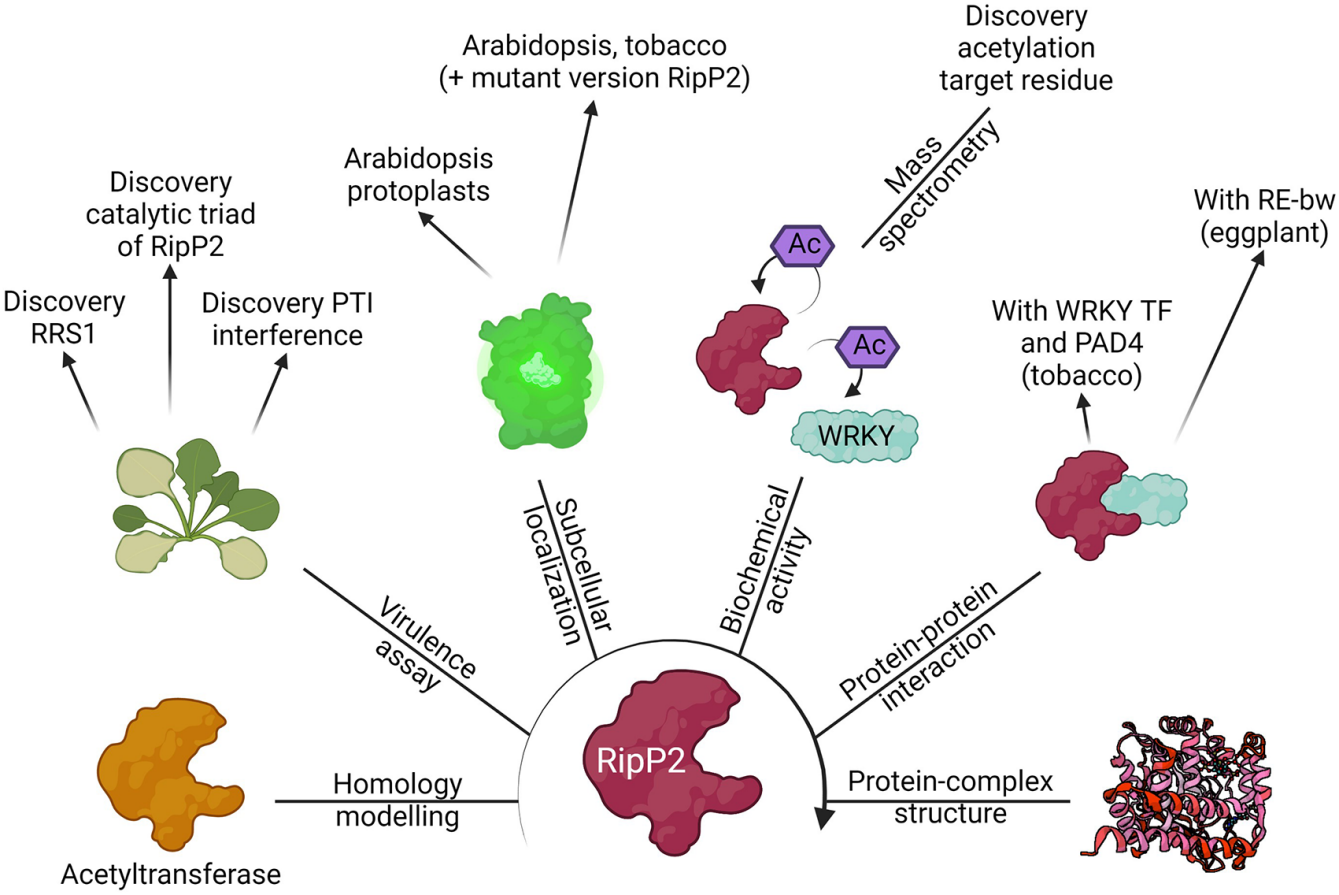
The route for the functional characterization of RipP2. Different laboratories have contributed to the elucidation of the putative function of the T3E RipP2, formally known as PopP2, by using the different methods discussed in this review. Starting from its homology with other known acetyltransferases, the biochemical activity of the T3E (acetylation of WRKY transcription factors, including RRS1) was found as well as the catalytic residues responsible for this function. Eventually, the apo crystal structure of RipP2 or RipP2 in complex with IP_6_, acetyl-CoA, and the WRKY domain from RRS1-R was determined. This figure provides an overview of the results found by Deslandes et al. (2003), Tasset et al. (2010), Le Roux et al. (2015), Xiou et al. (2015), Zhang et al. (2017), Xia et al. (2021), and Huh (2022).

In this review, the RSSC was used as a case study to exemplify the current advances made using both conventional and state-of-the-art experimental techniques that have helped to functionally characterize T3Es. An overview of the methodologies used to elucidate the function of RipP2, one of the best described T3Es from the RSSC, is illustrated (Figure 4).

Clearly, complementary discoveries add onto one another and accelerate the functional characterization of a protein. In the case of RipP2, the homology with acetyltransferases and the link with the RRS1-R protein in *Arabidopsis* has led to the eventual uncovering of the trans- and autoacetylation function of RipP2 (Figure 4; Deslandes et al., 2003; Tasset et al., 2010). This finding was mined for further identification of the RipP2 catalytic triad, for mapping the acetyl-receiving residues of host WRKY transcription factors, and elucidation of the apo crystal structure of RipP2 and RipP2 in complex with IP_6_, acetyl-CoA, and the WRKY domain from RRS1-R (Le Roux et al., 2015; Xiou et al., 2015; Zhang et al., 2017; Xia et al., 2021). In turn, available structural data might be helpful for homology modelling and functional characterization of a different effector protein.

Regarding the RSSC, still much research is required to fully functionally characterize its effectome. As mentioned, this process is hindered by the effectome size, the strain diversity, and the broad host range of the pathogen. Ample efforts were undertaken to optimize T3E prediction and to understand its effect on the HR response, for instance by tobacco infiltration of an effectorless mutant strain. Further, increasing knowledge on RSSC effector functioning could be obtained by performing complementary transcriptome, proteome, or metabolome studies in different host plants. The exact functions and host targets of various T3Es remain unsolved, although multiple T3Es from the RSSC have a (predicted) enzymatic function: RipE1 has a protease activity, targeting JAZ proteins; RipG T3E family members mimic eukaryotic E3 ligases to target unknown host proteins for degradation; RipAR, RipAW, and RipV2 have evolved novel E3 ubiquitin ligase domains and RipN is a nudix hydrolase which can hydrolyze a wide range of organic pyrophosphates (for a review, see Schreiber et al., 2021).

To elucidate their (enzymatic) targets, the need for specialized methods is high. For the identification of protease substrates, for example, a “degradomics” approach could be opted. This subfield of proteomics compares proteomes with and without proteolytic activity, but this approach, remains to be explored in the context of effector-plant interactions (Mooney et al., 2021). It would also be interesting to study whether T3Es behave differently, not only in different hosts, but also at various stages, or even in different environmental niches during infection. To this end, a time-course (multi)-omics experiment for different hosts could be envisaged, but undoubtedly in the most natural environment as possible for the different host infections. By native delivery of a tagged T3E upon infection, for example, the EH-PPI network exploited by the T3E could be investigated at different timepoints and/or in different hosts. As effectors seem to function in tightly connected networks, information on their potential cooperativity in the plant cell could also improve our understanding of bacterial pathogen-mediated disease development in plants (Sanchez-Garrido et al., 2021). Finally, collaborative efforts of the scientific community will allow the full functional description of the effectome and help understand the disease in general.

It is important to remain vigilant when characterizing a T3E, because new viewpoints and techniques could modify the perception of previous work. Techniques previously mainly used in mammalian cells, such as BioID, are applied to the plant field and have more recently successfully been used for the functional characterization of T3Es (Khan et al., 2018; González-Fuente et al., 2020). Technology has also progressed, such as the development of new BioID modules (e.g., TurboID and ultraID) that allow the use of these methods in broader and more natural (*in vivo*) settings (Branon et al., 2018; Kubitz et al., 2022). Still, scientists should always be on the lookout for generation of new experimental techniques that can overcome (part of) the shortcomings of others. The use of single-cell omics profiling, for example, is already being used for plant systems and a single-cell omics perspective of T3E research would certainly result in a more detailed comprehension of the disease (Clark et al., 2022), because different host cell types might respond differently to the pathogen or to its deployed effectome. As *R. solanacearum* moves through distinct cell types of the plant (Planas-Marquès et al., 2020), single-cell omics profiling could also be used in a similar way to look at T3E expression and translocation, thereby more accurately distinguishing early from late T3E functioning. Altogether, the insights gained into T3E functioning by complementary approaches (e.g., AP-MS and BioID) should always be explored and combined to obtain a more detailed understanding of T3E functioning.

## Author contributions

JDR: conceptualization, investigation, figure preparations, and writing—review and editing. PVD: supervision, conceptualization, investigation, figure preparations, and writing—review and editing. SG: supervision, conceptualization, investigation, resources, and writing—review and editing. All authors contributed to the article and approved the submitted version.

## Funding

This work was supported by the European Research Council (ERC) under the European Union’s Horizon 2020 research and innovation program (PROPHECY grant agreement No 803972) from the Research Foundation—Flanders (project number G.0511.20N to PVD) and by the Special Research Fund – Concerted Research Actions (project number BOF18-GOA-013 to SG). JDR is a predoctoral fellow of the Research Foundation-Flanders (Strategic Basic Research fellowship no. 1S83919N).

## Conflict of interest

The authors declare that the research was conducted in the absence of any commercial or financial relationships that could be construed as a potential conflict of interest.

## Publisher’s note

All claims expressed in this article are solely those of the authors and do not necessarily represent those of their affiliated organizations, or those of the publisher, the editors and the reviewers. Any product that may be evaluated in this article, or claim that may be made by its manufacturer, is not guaranteed or endorsed by the publisher.

## Footnotes

^1^ https://iant.toulouse.inra.fr/bacteria/annotation/site/prj/T3Ev3/

^2^ www.effectorK.org
