# Supplementary material for: From prediction to function: Current practices and challenges towards the functional characterization of type III effectors
Source: Front Microbiol. 2023 Feb 8;14:1113442. doi: 10.3389/fmicb.2023.1113442 (PMC9945535; doi:10.3389/fmicb.2023.1113442)
Supplement: Supplementary file 1 [file Table_1.docx]

Supplementary Table 1. Characterized T3Es from the *R. solanacearum* species complex with known *in planta* functions. T3Es with at least one known function are listed as well as the methods that led to the discovery of this function and that are relevant for the purpose of this review.

| T3E | Function | Method | Goal | Model | Reference |
| --- | --- | --- | --- | --- | --- |
| RipA5 | Inhibitor of TOR signaling | Yeast inhibition assay^1^ | Identification of T3Es inhibiting yeast growth inhibition. | Yeast | Popa et al. (2016) |
|  |  | Transcriptome analysis (DNA microarray)^1^ | Time-course experiment to test the changes on mRNA levels by RipA5 | Yeast |  |
|  |  | Yeast inhibition assay^2^ | Test for the influence of expression of TOR-related genes on the RipA5-related yeast growth inhibition phenotype. | Yeast |  |
|  |  | Transcriptome analysis (RNA-seq)^1^ | Complementary method to the DNA microarray experiment. | Yeast |  |
|  |  | Autophagy assay^2^ | Test for the influence of RipA5 expression on autophagy. | Yeast |  |
|  |  | Nitrate reductase activity assay^2^ | Test for the effect of RipA5 on nitrate reductase activity. | Tobacco |  |
|  |  | Virulence assay^2^ | Test for the influence of TOR silencing on disease symptom development. | *Arabidopsis* |  |
| RipAB | Interference with Ca^2+^‐dependent gene expression | Virulence screening^1^ | Revelation of the virulence contribution of core effectors. | Potato, tobacco, yeast | Zheng et al. (2019) |
|  |  | Confocal microscopy^2^ | *In planta* localization of RipAB. | Tobacco |  |
|  |  | Transcriptome analysis (RNA-seq)^1^ | Effect of RipAB on plant processes. | Potato |  |
|  |  | qRT-PCR^2^ | Validation of RNA-seq data. | Potato |  |
|  |  | Virulence assay^2^ | ROS measurement. | Potato |  |
|  | Interference with salicylic acid signalling by targeting TGA transcription factors | Virulence assay^1^ | Screen for T3Es containing nuclear localization signals that affect plant immune gene expression. | *Arabidopsis* protoplasts, *Arabidopsis* | Qi et al. (2022) |
|  |  | Virulence assay^2^ | Test for the effect of RipAB on ROS induction and MAPK activation. | Tobacco, *Arabidopsis* protoplasts, *Arabidopsis* |  |
|  |  | Confocal microscopy^2^ | *In planta* localization of RipAB. | *Arabidopsis* protoplasts and *Arabidopsis* |  |
|  |  | Virulence assay | Test for RipAB overexpression or deletion resulting in more severe disease symptoms. | *Arabidopsis*, tomato |  |
|  |  | IP-MS^1^ | Screen for plant protein interactors of RipAB. | Tobacco |  |
|  |  | BiFC, split-LUC, co-IP^2^ | Validation of the IP-MS data. | Tobacco |  |
|  |  | GST/MBP pull-down assay^2^ | Determination of direct or indirect binding of RipB with TGA TFs | *In vitro* |  |
|  |  | Virulence assay | Test for RipAB influence on SA-induced resistance and on RipAB virulence dependence on TGAs. | *Arabidopsis* |  |
|  |  | Transcriptome analysis (RNA-seq)^1^ | Investigation of the influence of RipAB expression on SA-mediated gene expression. | *Arabidopsis* |  |
|  |  | RT-qPCR^2^ | Validation of the RNA-seq data. | *Arabidopsis* |  |
|  |  | ChIP-PCR^1^ | Search for the transcriptional targets of TGAs in the presence or absence of RipAB. | *Arabidopsis* protoplasts |  |
|  |  | Co-IP^2^ | Test for RipAB interference with NPR1-TGA2 or with TGA2-RNA polymerase II protein interaction. | *Arabidopsis* protoplasts |  |
|  |  | ChIP-qPCR^2^ | Test for RipAB interference with the ability of TGAs to recruit RNA polymerase II to the *PR1* promoter. | *Arabidopsis* |  |
| RipAC | Suppression of NLR-mediated SGT1-dependent immune responses. | Virulence assay^1^ | Effect of RipAC on wilting and bacterial multiplication in xylem sap. | *Arabidopsis*, tomato | Yu et al. (2020) |
|  |  | IP-MS^1^ | Screen for plant protein interactors of RipAC. | Tobacco |  |
|  |  | Co-IP, split-LUC, split-YFP^2^ | Validation of IP-MS data. | Tobacco |  |
|  |  | Virulence assay^2^ | Effect of RipAC on HR and on MAPK activation. | Tobacco |  |
|  |  | IP-MS^2^ | Effect of RipAC on PTMs of SGT1. | Tobacco |  |
|  |  | IP^2^ | Effect of RipAC on SGT1 phosphorylation and MAPK3/4/6-SGT1 interaction. | *Arabidopsis*, tobacco |  |
|  |  | Virulence assay^2^ | Effect of SGT1 overexpression on wilting caused by WT or *ΔripAC* mutant. | *Arabidopsis* |  |
|  | Targets SGT1 to suppress ETI. | Virulence assay^1^ | Effect of several T3Es on RipAA-induced HR. | Tobacco | Nakano et al., (2021) |
|  |  | Virulence assay^2^ | Test the requirement of the LRR domain of RipAC on RipAA- and RipP1-induced HR. | Tobacco |  |
|  |  | Confocal microscopy^1^ | Subcellular localization. | Tobacco |  |
|  |  | Y2H-Seq^1^ | Screen for tobacco protein interactors of RipAC. | Yeast |  |
|  |  | Split-luciferase^2^ | Validation of Y2H-Seq and to test the effect of RipAC expression on the SGT1-RAR1 interaction. | Tobacco |  |
|  |  | Virulence assay^2^ | Test the influence of *ripAC* mutation on *R. solanacearum* growth *in planta.* | Tobacco |  |
|  | Targets plant E3 ubiquitin ligase PUB4 | Virulence assay^1^ | Effect of RipAC-GFP expression on ROS and MAPK accumulation, as well as response to an (a)virulent *P. syringae* DC3000 strain. | *Arabidopsis* | Yu et al., (2022) |
|  |  | Y2H-Seq^1^ | Screen for tomato protein interactors of RipAC. | Yeast |  |
|  |  | Split-luciferase, co-IP, FRET-FLIM^2^ | Validation of Y2H-Seq. | Tobacco |  |
|  |  | Co-IP^2^ | Test the effect of RipAC on PUB4 and PRR accumulation. | *Arabidopsis* |  |
|  |  | IP-MS^2^ | Test the effect of RipAC on PUB4 accumulation after PAMP treatment. | *Arabidopsis* |  |
| RipAF1 | ADP-ribosylation of host fibrillin FBN1. | Virulence assay^1^ | Test the effect of RipAF1 expression on tobacco leaf chlorosis and disease development. | Tobacco | Wu et al., (2022) |
|  |  | qRT-PCR^2^ | Determine the effect of RipAF1 on the JA and SA signaling pathways. | Tobacco |  |
|  |  | Y2H-Seq^1^ | Screen for tobacco protein interactors of RipAF1. | Yeast |  |
|  |  | MBP pull down assay, split-luciferase^2^ | Validation of the Y2H-Seq results. | Tobacco |  |
|  |  | Confocal microscopy^1^ | Subcellular localization. | Tobacco leaf and protoplasts |  |
|  |  | Biochemical activity assay^2^ | Test the mADP-RT activity of RipAF1. | *E. coli* |  |
|  |  | Split-luciferase, BiFC, qRT-PCR^2^ | Test the influence of loss of mADP-RT activity on interaction with NbFBN1. |  |  |
|  |  | IP-MS^2^ | Identify the target residues of RipAF1. | *E. coli* |  |
|  |  | Co-IP^2^ | Validation of the IP-MS results. | Tobacco |  |
| RipAK | Interaction with and inhibition of the activity of host catalases | Virulence assay^1^ | Effect of RipAK on HR, multiplication and ROS accumulation. | Tobacco | Sun et al. (2017) |
|  |  | Confocal microscopy^2^ | Subcellular localization. | *Arabidopsis* protoplasts |  |
|  |  | IP^1^ | Screen for plant protein interactors of RipAK. | *Arabidopsis* |  |
|  |  | Y2H, BiFC^2^ | Validation of IP data. | Yeast, *Arabidopsis* protoplasts |  |
|  |  | Catalase activity assay^2^ | Test for the influence of RipAK on catalase activity. | Yeast, tobacco |  |
|  |  | Transcriptome analysis (RNA-seq)^1^ | Effect of RipAK on the global host transcriptional response. | Tobacco |  |
|  |  | qRT-PCR^2^ | Validation of RNA-seq data. | Tobacco |  |
|  | Interaction with and inhibition of host pyruvate decarboxylases. | Virulence assay^1^ | Effect of a *ripAK* mutant on virulence. | *Arabidopsis*, tomato | Wang et al., (2021) |
|  |  | Confocal microscopy^2^ | Subcellular localization. | Tobacco, *Arabidopsis* protoplasts |  |
|  |  | Y2H-Seq^1^ | Screen for tomato root protein interactions with RipAK. | Yeast |  |
|  |  | Co-localization, co-IP, split-luciferase^2^ | Validation of the Y2H-Seq results. | Tobacco |  |
|  |  | Virulence assay^2^ | Effect of *ripAK* and/or *pdc2* mutant on virulence. | *Arabidopsis* |  |
|  |  | Biochemical activity assay^2^ | Test the effect of RipAK on the enzymatic activity and accumulation of PDC2. | Tobacco, *Arabidopsis* |  |
|  |  | Split-luciferase^2^ | Test the effect of RipAK on the oligomerization of AtPDC2. | Tobacco |  |
| RipAY | Association with plant h-type thioredoxins and degradation of glutathione *in planta* to interfere with immune responses. | Virulence assay^1^ | Effect of RipAY on ROS accumulation. | Tobacco | Sang et al. (2018) |
|  |  | qRT-PCR^2^ | Effect of RipAY on SA-dependent responses. |  |  |
|  |  | Confocal microscopy^1^ | Subcellular localization. |  |  |
|  |  | IP-MS^1^ | Screen for plant protein interactors of RipAY. |  |  |
|  |  | Co-IP^2^ | Validation of IP-MS data. |  |  |
|  |  | Expression assays^2^ | Silencing and overexpression of thioredoxins and its effect on ROS accumulation. |  |  |
|  |  | Glutathione measurement^2^ | Investigation of the ability of RipAY to degrade glutathione |  |  |
|  |  | Virulence assay | Effect of an amino acid substitution on the ability of RipAY to induce ROS or alter SA-related expression. |  |  |
|  |  | Yeast inhibition assay^1^ | Identification of T3Es causing yeast growth inhibition. | Yeast | Fujiwara et al. (2016, 2020) |
|  |  | Fluorescence microscopy^2^ | Localization of RipAY in yeast cells. | Yeast |  |
|  |  | GGCT activity assay^2^ | Test for GGCT activity of RipAY toward glutathione. | Yeast |  |
|  |  | Yeast inhibition assay^2^ | Test for the GGCT activity being responsible for yeast growth inhibition and for the alteration of the phenotype by glutathione addition. | Yeast |  |
|  |  | Glutathione measurement^2^ | Test for the effect of RipAY on plant glutathione levels | Eggplant |  |
|  |  | Biochemical assay^1^ | Identification of the eukaryotic activator for GGCT activity of RipAY. | Yeast |  |
|  |  | Y2H^2^ | Test for the protein interaction between RipAY and thioredoxins. | Yeast |  |
|  |  | Kinetic analysis^2^ | Investigation of the catalytic activity of RipAY. | Yeast |  |
|  |  | Stoichiometric analysis^2^ | Verification of the stoichiometry of the RipAY-thioredoxin complex. | *E. coli* |  |
|  | Suppression of the RipE1-triggered immune response | Virulence assay^2^ | Cell death assay and ion leakage assay to study the relationship between RipAY and RipE1. | Tobacco | Sang et al. (2020) |
|  |  | qRT-PCR^2^ | Test for the effect of RipAY on SA-related genes induced by RipE1. | Tobacco |  |
| RipB | Contributes to virulence and interferes with ROS production and cytokinin pathways. | Virulence assay^2^ | ROS assay and cell death assay to test the effect of RipB expression in tobacco leaves. | Tobacco | Cao et al., (2022) |
|  |  | Virulence assay^1^ | Test the effect of *ripB* expression on *in planta* bacterial multiplication and virulence. | *Arabidopsis* |  |
|  |  | qRT-PCR^2^ | Test the effect of *ripB* expresiion on cytokinin-related gene expression. | *Arabidopsis* |  |
|  |  | Y2H^2^ | Test the interaction of RipB with Arabidopsis 14-3-3 proteins. | Yeast |  |
| RipE1 | Interference with jasmonate signaling. | Virulence assay^1^ | ROS assay to screen *Ralstonia* T3Es that suppress Flg22-triggered ROS burst. | Tobacco | Nakano and Mukaihara (2019) |
|  |  | Protease assay | Test for the ability of RipE1 to manipulate and activate jasmonate signaling by cysteine protease activity. | Tobacco |  |
|  |  | Virulence assay^2^ | Ion leakage assay for RipE1. | Tobacco |  |
|  |  | Confocal microscopy^2^ | Subcellular localization. | Tobacco |  |
|  |  | Protein-protein interaction^1^ | Y2H and split-YFP to investigate interaction of RipE1 with JAZ proteins. | Yeast, tobacco |  |
|  |  | qRT-PCR^2^ | Test for the effect of RipE1 on JA and SA signaling. | Tobacco |  |
|  |  | Virulence assay^2^ | RipE1 overexpression assay to study its role in disease symptom development in relation to JA. | *Arabidopsis* |  |
|  |  | Virulence assay^1^ | Cell death assay and ion leakage assay of RipE1 to study its effect on tobacco. | Tobacco | Sang et al. (2020) |
|  |  | qRT-PCR^2^ | Study of the effect of RipE1 on SA- and JA-related gene expression. | Tobacco, *Arabidopsis* |  |
|  |  | Virulence assay^2^ | Study of the effect of RipE1-experssion on disease development. | *Arabidopsis* |  |
|  | Interaction with and cleaving of the *Arabidopsis* Exo70B1. | Y2H^1^ | Screen for protein interactions between RipE1 or a RipE1 mutant form and NLR-IDs. | *Yeast* | Tsakiri et al., (2022) |
|  |  | Virulence assay^1^ | Screen for RipE1-triggered HR response in different *Nicotiana* species. | Tobacco |  |
|  |  | Confocal microscopy^2^ | Colocalization of RipE1 and Exo70B1. | Tobacco |  |
|  |  | Co-IP^2^ | Validation of the RipE1-Exo70B1 interaction. | Tobacco |  |
|  |  | Biochemical activity assay^2^ | Cleavage assays with the purified proteins of RipE1 and Exo70B1. Confirmation of cleavage by MS. | *In vitro*, tobacco |  |
|  |  | Virulence assay^2^ | Influence of RipE1 expression paired with Exo70B1 and/or TN2 expression on HR. | *Tobacco* |  |
| RipI | Enhances the production of GABA to support bacterial nutrient acquisition during plant infection. | Virulence assay^1^ | Test the effect of a *ripI* mutant on virulence (disease symptoms, bacterial multiplication). | *Arabidopsis*, tomato | Xian et al., (2020) |
|  |  | IP-MS^1^ | To identify plant targets of RipI-GFP. | Tobacco |  |
|  |  | Y2H-Seq^1^ | To identify tomato targets of RipI. | Yeast |  |
|  |  | Co-IP, FRET-FLIM^2^ | Validation of protein interactions. | Tobacco |  |
|  |  | LC-MS^2^ | Test the effect of RipI expression on GABA accumulation. | Tobacco, *Arabidopsis* |  |
|  |  | CaM affinity pull-down | Test the effect of RipI expression on GAD accumulation. | *Arabidopsis* |  |
|  |  | Virulence assay^2^ | Test the effect of *ripI* mutant on virulence in *gad1/2* mutant plants. | *Arabidopsis* |  |
|  | Induces host defense by interaction with a bHLH93 transcription factor. | Virulence assay^1^ | The effect of RipI on HR and disease development. | Tobacco, tomato | Zhuo et al., (2020) |
|  |  | Y2H-Seq^1^ | Screen for tobacco protein interactors with RipI.GST pull-down was used as validation. | Yeast |  |
|  |  | Confocal microscopy^1^ | Subcellular localization of RipI and bHLH93. | *Arabidopsis* protoplasts |  |
|  |  | Virulence assay^2^ | Gene silencing of *bHLH93* and effect on RipI-induced HR. Validation by qRT-PCR. | Tobacco |  |
| RipN | Suppression of PTI and alteration of NADH/NAD^+^ levels in *Arabidopsis.* | Nudix hydrolase enzymatic assay^1^ | Test for RipN ability to hydrolyze diverse substrates. |  | Sun et al. (2019) |
|  |  | Substrate measurements^2^ | Validation of the results from the enzymatic assay. | *Arabidopsis* |  |
|  |  | Virulence assay^2^ | The effect of RipN expression on i*n planta* bacterial growth and callose deposition. | *Arabidopsis* |  |
|  |  | Confocal microscopy | Subcellular localization. | Tobacco, *Arabidopsis* protoplasts |  |
|  |  | ELISA and western blotting assay | Test for the influence of RipN on the amounts of ADP-ribosylated and poly-ADP-ribosylated proteins. | *Arabidopsis* |  |
| RipP2 | Interaction with the R protein RRS1-R. | Virulence assay^1^ | The effect of RipP2 on wilting and *in planta* bacterial growth. | *Arabidopsis* | Deslandes et al. (2003) |
|  |  | Protein-protein interaction^1^ | Split-ubiquitin analysis to test the interaction between RipP2 and RRS1-R. | Yeast |  |
|  |  | Confocal microscopy^2^ | Subcellular localization of RipP2 and RRS1-R. | *Arabidopsis* protoplasts |  |
|  | Autoacetylation of a lysine residue required for RRS1-R-mediated immunity. | Virulence assay^2^ | Test for the effect of a mutation in the catalytic triad on the function of PopP2 during infection. | *Arabidopsis* | Tasset et al. (2010) |
|  |  | Confocal microscopy^2^ | Subcellular localization of RipP2 and the mutant version. | *Arabidopsis*, tobacco |  |
|  |  | FRET-FLIM^2^ | Validation of the confocal microscopy results. | *Arabidopsis*, tobacco |  |
|  |  | Co-expression assay | Measurement of protein accumulation levels of RRS1-R, RRS1-S and RipP2. | Tobacco |  |
|  |  | Enzymatic activity assay^1^ | Test for the auto-acetylation and acetyl-transferase capacity of RipP2. | *E. coli* |  |
|  | Acetylation of WRKY-TF to disrupt TF-DNA interaction | Immunoblot assay | Test for the ability of RipP2 to acetylate RRS1-R. | Tobacco | Le Roux et al. (2015) |
|  |  | MS proteomic analysis^2^ | Identification of the modifications on RRS1-R residues by RipP2. | Tobacco |  |
|  |  | Bacterial acetylation assay | Test for the acetylation of the RRS1-R WRKY-domain by RipP2. | *E. coli* |  |
|  |  | IP-MS^2^ | Validation of the results obtained from the bacterial acetylation assay. | *E. coli* |  |
|  |  | Protein-DNA FRET-FLIM and EMSA | Test for the effect of RRS1-R acetylation to its DNA-binding capacity. | Tobacco, *E. coli* |  |
|  |  | Co-IP^1^ | Test for the interaction between RipP2 and other WRKY transcription factors. | Tobacco |  |
|  |  | Protein-DNA FRET-FLIM^2^ | Validation of the Co-IP results. | Tobacco |  |
|  |  | Virulence assay^2^ | Root infection assay to test the contribution of RipP2 to virulence. | *Arabidopsis* |  |
|  |  | Virulence assay^2^ | Test for RipP2 interference with PTI. | Tobacco |  |
|  |  | qRT-PCR^2^ | Test for the effect of active RipP2 on flg22-induced gene expression. | Tobacco |  |
|  | Binding with the putative resistance gene *RE-bw* in eggplant. | Protein-protein interaction^1,2^ | BiFC and Y2H assay to test the interaction between RE-bw and RipP2. | Eggplant protoplasts, yeast | Xi'ou et al. (2015) |
|  | Structural evidence for the RipP2-RRS1-R_WRKY_ interaction. | Protein crystallization | Determination of the crystal structure of RipP2 (catalytic mutant) in complex with IP_6_, AcCoA and the RRS1-R_WRKY_ domain_._ | *In vitro* | Zhang et al. (2017) |
|  |  | NMR and isothermal titration calorimetry | Investigation of the effect of IP_6_ binding on the RipP2-CoA interaction. | *In vitro* |  |
|  |  | Acetylation assay^2^ | Study of the effect of IP_6_ on the acetylation potential of RipP2. | *In vitro* |  |
|  |  | Virulence assay^2^ | Test for the effect of mutations in the catalytic site of RipP2 on the ability to induce HR. | *Arabidopsis* (ecotype Nd-1) |  |
|  | Crystal structure of apo RipP2 | Protein crystallization | Determination of the apo crystal structure of RipP2 | *In vitro* | Xia et al. (2021) |
|  | Interacts with PAD4 in an acetyl-transferase activity-dependent manner. | Co-IP^2^ | Test the interaction between RipP2 and PAD4, SAG101 or EDS1, as well as the influence of a catalytic RipP2 mutant. Validation by BiFC. | Tobacco | Huh, (2022) |
|  |  | Acetylation assay^2^ | Study the acetylation of PAD4 or EDS1 by RipP2. | Tobacco |  |
|  |  | Virulence assay^1^ | Test the effect of RipP2 on *Arabidopsis* mutants | *Arabidopsis* |  |
| RipTAL | Activation of *ADC* genes to boost host polyamine levels. | Promoter-reporter assay^1^ | Search for effector-binding elements (EBEs) | Tobacco | Wu et al. (2019) |
|  |  | qRT-PCR^2^ | Test for the influence of RipTAL expression on host *ADC* mRNA levels. | Tomato, tobacco, eggplant |  |
|  |  | Transcriptome analysis (RNA-seq)^1^ | Search for other RipTAL target genes. | Tomato |  |
|  |  | 5’ rapid amplification of cDNA ends (RACE)^1^ | Determination of the influence of RipTAL on the 5’ UTR of *ADC* transcripts. | Tomato, tobacco, eggplant |  |
|  |  | Translational activity assay^2^ | Test for the effect of different 5’ UTRs on GFP levels. | Tobacco, tobacco cell lysates |  |
|  |  | MS-based assay^2^ | Test for the effects of RipTAL on ADC activity and on metabolite compositions. | Tomato |  |
|  |  | Virulence assay^2^ | Test for the effect of RipTAL and ADCs on pathogen growth | Tomato leaf |  |
|  |  | 5’ RACE-PCR and MS-based assay^2^ | Test for the effect of RipTAL and ADC levels and ADC activity. | Tomato root |  |
| RipTPS | Management of production of plant trehalose-6-phosphate | CyaA translocation assay^2^ | Confirmation of the translocation of RipTPS into host cells. | Tobacco | Poueymiro et al. (2014) |
|  |  | Yeast inhibition assay^2^ | Test for complementation of RipTPS in a *tps1 tps2* yeast double mutant. | Yeast |  |
|  |  | Trehalose-6-phosphate measurement^2^ | Test for the effect of RipTPS and mutant forms on trehalose-6-phosphate production. | Yeast |  |
|  |  | Virulence assay^1^ | Test for the effect of RipTPS or a mutant form on disease symptom development of different host plants. | *Arabidopsis*, tomato, eggplant, geranium, bean |  |
|  |  | Virulence assay^2^ | Test for the effect of RipTPS or a mutant form lacking the C-terminal half on HR-like necrotic responses. | Tobacco |  |
| RipX | Suppression of the mitochondrial *atpA* gene. | Virulence assay^1^ | Test for the effect of RipX1 and RipX3 expression on leaf chlorosis and HR. | Tobacco | Sun et al., (2020) |
|  |  | qRT-PCR^2^ | Monitor the expression levels of defense-related genes. | Tobacco |  |
|  |  | Y2H-Seq^1^ | Screen for tobacco protein interactors with RipX1. | Yeast |  |
|  |  | Split-luciferase, BiFC^2^ | Validation of the Y2H-Seq data. | Tobacco |  |
|  |  | Confocal microscopy^1^ | Colocalization of RipX1 with ATPA. | Tobacco |  |
|  |  | VIGS^2^ | Test the effect of *atpA* silencing on disease development. | Tobacco |  |
|  |  | qRT-PCR, promoter-GUS fusion^2^ | Monitor the expression level of *atpA* in response to RipX1. | Tobacco |  |

^1^screening method, ^2^validation method.

**References**

Cao, P., Chen, J., Wang, R., Zhao, M., Zhang, S., An, Y., Liu, P., & Zhang, M. (2022). A conserved type III effector RipB is recognized in tobacco and contributes to Ralstonia solanacearum virulence in susceptible host plants. *Biochemical and Biophysical Research Communications*, *631*, 18–24. https://doi.org/10.1016/J.BBRC.2022.09.062

Deslandes, L., Olivier, J., Peeters, N., Feng, D. X., Khounlotham, M., Boucher, C., Somssich, I., Genin, S., & Marco, Y. (2003). Physical interaction between RRS1-R, a protein conferring resistance to bacterial wilt, and PopP2, a type III effector targeted to the plant nucleus. *Proceedings of the National Academy of Sciences of the United States of America*, *100*(13), 8024. https://doi.org/10.1073/PNAS.1230660100

Fujiwara, S., Ikejiri, A., Tanaka, N., & Tabuchi, M. (2020). Characterization of the mechanism of thioredoxin-dependent activation of γ-glutamylcyclotransferase, RipAY, from Ralstonia solanacearum. *Biochemical and Biophysical Research Communications*, *523*(3), 759–765. https://doi.org/10.1016/J.BBRC.2019.12.092

Fujiwara, S., Kawazoe, T., Ohnishi, K., Kitagawa, T., Popa, C., Valls, M., Genin, S., Nakamura, K., Kuramitsu, Y., Tanaka, N., & Tabuchi, M. (2016). *RipAY, a Plant Pathogen Effector Protein, Exhibits Robust γ-Glutamyl Cyclotransferase Activity When Stimulated by Eukaryotic Thioredoxins*. *291*(13). http://www.ncbi.nlm.nih.gov/pubmed/26823466

Huh, S. U. (2022). PopP2 interacts with PAD4 in an acetyltransferase activity-dependent manner and affects plant immunity. *Https://Doi.Org/10.1080/15592324.2021.2017631*, *16*(12). https://doi.org/10.1080/15592324.2021.2017631

Le Roux, C., Huet, G., Jauneau, A., Camborde, L., Trémousaygue, D., Kraut, A., Zhou, B., Levaillant, M., Adachi, H., Yoshioka, H., Raffaele, S., Berthomé, R., Couté, Y., Parker, J. E., & Deslandes, L. (2015). A Receptor Pair with an Integrated Decoy Converts Pathogen Disabling of Transcription Factors to Immunity. *Cell*, *161*(5), 1074–1088. https://doi.org/10.1016/J.CELL.2015.04.025

Nakano, M., Ichinose, Y., & Mukaihara, T. (2021). Ralstonia solanacearum Type III Effector RipAC Targets SGT1 to Suppress Effector-Triggered Immunity. *Plant and Cell Physiology*, *61*(12), 2067–2076. https://doi.org/10.1093/PCP/PCAA122

Nakano, M., & Mukaihara, T. (2019). Comprehensive Identification of PTI Suppressors in Type III Effector Repertoire Reveals that Ralstonia solanacearum Activates Jasmonate Signaling at Two Different Steps. *International Journal of Molecular Sciences*, *20*(23). https://doi.org/10.3390/IJMS20235992

Popa, C., Li, L., Gil, S., Tatjer, L., Hashii, K., Tabuchi, M., Coll, N. S., Ariño, J., & Valls, M. (2016). The effector AWR5 from the plant pathogen Ralstonia solanacearum is an inhibitor of the TOR signalling pathway. *Scientific Reports 2016 6:1*, *6*(1), 1–14. https://doi.org/10.1038/srep27058

Poueymiro, M., Cazalé, A. C., François, J. M., Parrou, J. L., Peeters, N., & Genin, S. (2014). A Ralstonia solanacearum type III effector directs the production of the plant signal metabolite trehalose-6-phosphate. *MBio*, *5*(6). https://doi.org/10.1128/MBIO.02065-14/SUPPL_FILE/MBO006142098ST1.XLSX

Qi, P., Huang, M., Hu, X., Zhang, Y., Wang, Y., Li, P., Chen, S., Zhang, D., Cao, S., Zhu, W., Xie, J., Cheng, J., Fu, Y., Jiang, D., Yu, X., & Li, B. (2022). A Ralstonia solanacearum effector targets TGA transcription factors to subvert salicylic acid signaling. *The Plant Cell*. https://doi.org/10.1093/PLCELL/KOAC015

Sang, Y., Wang, Y., Ni, H., Cazalé, A. C., She, Y. M., Peeters, N., & Macho, A. P. (2018). The Ralstonia solanacearum type III effector RipAY targets plant redox regulators to suppress immune responses. *Molecular Plant Pathology*, *19*(1), 129. https://doi.org/10.1111/MPP.12504

Sang, Y., Yu, W., Zhuang, H., Wei, Y., Derevnina, L., Yu, G., Luo, J., & Macho, A. P. (2020). Intra-strain Elicitation and Suppression of Plant Immunity by Ralstonia solanacearum Type-III Effectors in Nicotiana benthamiana. *Plant Communications*, *1*(4), 100025. https://doi.org/10.1016/j.xplc.2020.100025

Sun, T., Wu, W., Wu, H., Rou, W., Zhou, Y., Zhuo, T., Fan, X., Hu, X., & Zou, H. (2020). Ralstonia solanacearum elicitor RipX Induces Defense Reaction by Suppressing the Mitochondrial atpA Gene in Host Plant. *International Journal of Molecular Sciences 2020, Vol. 21, Page 2000*, *21*(6), 2000. https://doi.org/10.3390/IJMS21062000

Sun, Y., Li, P., Deng, M., Shen, D., Dai, G., Yao, N., & Lu, Y. (2017). The Ralstonia solanacearum effector RipAK suppresses plant hypersensitive response by inhibiting the activity of host catalases. *Cellular Microbiology*, *19*(8), e12736. https://doi.org/10.1111/CMI.12736

Sun, Y., Li, P., Shen, D., Wei, Q., He, J., & Lu, Y. (2019). The Ralstonia solanacearum effector RipN suppresses plant PAMP‐triggered immunity, localizes to the endoplasmic reticulum and nucleus, and alters the NADH/NAD+ ratio in Arabidopsis. *Molecular Plant Pathology*, *20*(4), 533. https://doi.org/10.1111/MPP.12773

Tasset, C., Bernoux, M., Jauneau, A., Pouzet, C., Briére, C., Kieffer-Jacquinod, S., Rivas, S., Marco, Y., Deslandes, L., Brière, C., Kieffer-Jacquinod, S., Rivas, S., Marco, Y., & Deslandes, L. (2010). Autoacetylation of the Ralstonia solanacearum Effector PopP2 Targets a Lysine Residue Essential for RRS1-R-Mediated Immunity in Arabidopsis. *PLoS Pathogens*, *6*(11), e1001202. http://www.ncbi.nlm.nih.gov/pubmed/21124938

Tsakiri, D., Kotsaridis, K., Michalopoulou, V. A., Kokkinidis, M., & Sarris, P. F. (2022). Ralstonia solanacearum core effector RipE1 interacts and cleaves the Arabidopsis exocyst component Exo70B1. *BioRxiv*, 2022.08.31.506019. https://doi.org/10.1101/2022.08.31.506019

Wang, Y., Zhao, A., Morcillo, R. J. L., Yu, G., Xue, H., Rufian, J. S., Sang, Y., & Macho, A. P. (2021). A bacterial effector protein uncovers a plant metabolic pathway involved in tolerance to bacterial wilt disease. *Molecular Plant*, *14*(8), 1281–1296. https://doi.org/10.1016/J.MOLP.2021.04.014

Wu, D., von Roepenack-Lahaye, E., Buntru, M., de Lange, O., Schandry, N., Pérez-Quintero, A. L., Weinberg, Z., Lowe-Power, T. M., Szurek, B., Michael, A. J., Allen, C., Schillberg, S., & Lahaye, T. (2019). A Plant Pathogen Type III Effector Protein Subverts Translational Regulation to Boost Host Polyamine Levels. *Cell Host & Microbe*, *26*(5), 638-649.e5. https://doi.org/10.1016/J.CHOM.2019.09.014

Wu, W., Luo, X., Chen, X., Wang, L., Wang, K., Tian, S., Tong, Z., Zhao, T., Fan, X., Zhuo, T., Hu, X., & Zou, H. (2022). Ralstonia solanacearum effector RipAF1 ADP-ribosylates host FBN1 to induce resistance against bacterial wilt. *BioRxiv*, 2022.04.04.487053. https://doi.org/10.1101/2022.04.04.487053

Xia, Y., Zou, R., Escouboué, M., Zhong, L., Zhu, C., Pouzet, C., Wu, X., Wang, Y., Lv, G., Zhou, H., Sun, P., Ding, K., Deslandes, L., Yuan, S., & Zhang, Z. M. (2021). Secondary-structure switch regulates the substrate binding of a YopJ family acetyltransferase. *Nature Communications*, *12*(1). https://doi.org/10.1038/s41467-021-26183-1

Xian, L., Yu, G., Wei, Y., Xue, H., Morcillo, R. J. L., Correspondence, A. P. M., Rufian, J. S., Li, Y., Zhuang, H., & Macho, A. P. (2020). A Bacterial Effector Protein Hijacks Plant Metabolism to Support Pathogen Nutrition. *Cell Host and Microbe*, *28*, 548-557.e7. https://doi.org/10.1016/j.chom.2020.07.003

Xiou, X., Bihao, C., Guannan, L., Jianjun, L., Qinghua, C., Jin, J., & Yujing, C. (2015). Functional Characterization of a Putative Bacterial Wilt Resistance Gene (RE-bw) in Eggplant. *Plant Molecular Biology Reporter*, *33*(4), 1058–1073. https://doi.org/10.1007/S11105-014-0814-1/FIGURES/19

Yu, G., Derkacheva, M., Rufian, J. S., Brillada, C., Kowarschik, K., Jiang, S., Derbyshire, P., Ma, M., DeFalco, T. A., Morcillo, R. J. L., Stransfeld, L., Wei, Y., Zhou, J.-M., Menke, F. L. H., Trujillo, M., Zipfel, C., & Macho, A. P. (2022). The Arabidopsis E3 ubiquitin ligase PUB4 regulates BIK1 and is targeted by a bacterial type-III effector. *The EMBO Journal*, *41*(23), e107257. https://doi.org/10.15252/EMBJ.2020107257

Yu, G., Xian, L., Xue, H., Yu, W., Rufian, J. S., Sang, Y., Morcillo, R. J. L., Wang, Y., & Macho, A. P. (2020). A bacterial effector protein prevents MAPK-mediated phosphorylation of SGT1 to suppress plant immunity. *PLOS Pathogens*, *16*(9), e1008933. https://doi.org/10.1371/JOURNAL.PPAT.1008933

Zhang, Z. M., Ma, K. W., Gao, L., Hu, Z., Schwizer, S., Ma, W., & Song, J. (2017). Mechanism of host substrate acetylation by a YopJ family effector. *Nature Plants*, *3*(8), 17115. https://doi.org/10.1038/NPLANTS.2017.115

Zheng, X., Li, X., Wang, B., Cheng, D., Li, Y., Li, W., Huang, M., Tan, X., Zhao, G., Song, B., Macho, A. P., Chen, H., & Xie, C. (2019). A systematic screen of conserved Ralstonia solanacearum effectors reveals the role of RipAB, a nuclear‐localized effector that suppresses immune responses in potato. *Molecular Plant Pathology*, *20*(4), 547. https://doi.org/10.1111/MPP.12774

Zhuo, T., Wang, X., Chen, Z., Cui, H., Zeng, Y., Chen, Y., Fan, X., Hu, X., & Zou, H. (2020). The Ralstonia solanacearum effector RipI induces a defence reaction by interacting with the bHLH93 transcription factor in Nicotiana benthamiana. *Molecular Plant Pathology*, *21*(7), 999–1004. https://doi.org/10.1111/MPP.12937
